# Parahydrogen‐Induced Polarization in Hydrogenation Reactions Mediated by a Metal‐Free Catalyst

**DOI:** 10.1002/chem.202103501

**Published:** 2022-01-19

**Authors:** Danila O. Zakharov, Konstantin Chernichenko, Kristina Sorochkina, Shengjun Yang, Ville‐Veikko Telkki, Timo Repo, Vladimir V. Zhivonitko

**Affiliations:** ^1^ NMR Research Unit University of Oulu P.O. Box 3000 90014 Oulu Finland; ^2^ Department of Chemistry University of Helsinki A. I. Virtasen aukio 1 00014 Helsinki Finland; ^3^ Present address: Discovery, Product Development & Supply (DPDS) Janssen Pharmaceutical Companies of Johnson & Johnson Turnhoutseweg 30 2340 Beerse Belgium

**Keywords:** NMR hyperpolarization, Lewis acids, Lewis bases, organocatalysis, parahydrogen

## Abstract

We report nuclear spin hyperpolarization of various alkenes achieved in alkyne hydrogenations with parahydrogen over a metal‐free hydroborane catalyst (HCAT). Being an intramolecular frustrated Lewis pair aminoborane, HCAT utilizes a non‐pairwise mechanism of H_2_ transfer to alkynes that normally prevents parahydrogen‐induced polarization (PHIP) from being observed. Nevertheless, the specific spin dynamics in catalytic intermediates leads to the hyperpolarization of predominantly one hydrogen in alkene. PHIP enabled the detection of important HCAT‐alkyne‐H_2_ intermediates through substantial ^1^H, ^11^B and ^15^N signal enhancement and allowed advanced characterization of the catalytic process.

## Graphical abstract

**Substantial hyperpolarization** of nuclear spins in alkene products was observed in alkyne hydrogenations with parahydrogen mediated by a metal‐free hydroborane catalyst. The observed effects are unusual, as the mechanism of the reaction is not pairwise, thus allowing hyperpolarization of only one of two protons at double bonds. Moreover, this enabled various important intermediates to be characterized by hyperpolarized ^1^H, ^11^B and ^15^N NMR spectroscopy.

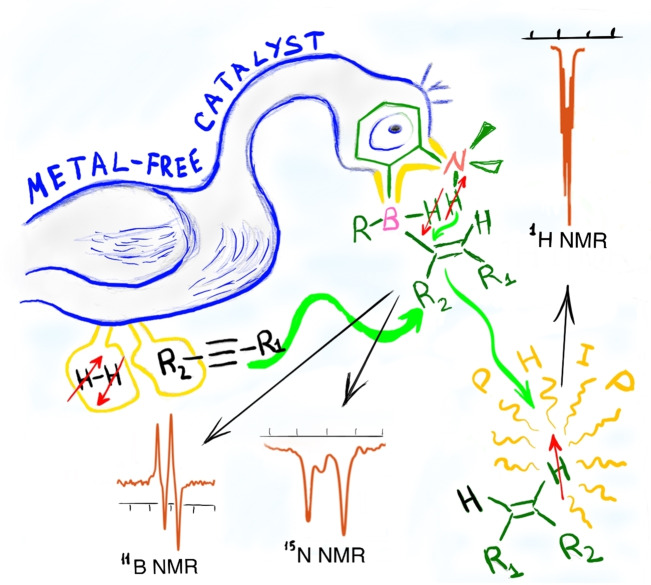

Nuclear magnetic resonance provides versatile tools for structure elucidation and understanding dynamics at the molecular level.^[1]^ It is also at the heart of magnetic resonance imaging (MRI), a non‐invasive and highly informative imaging method. However, the low intrinsic sensitivity of NMR dictated by low thermal spin polarization often leads to the insufficient signal strengths to perform reliable analysis. To date, several spin hyperpolarization techniques have been developed to boost the sensitivity.^[2]^ Among them, parahydrogen‐induced polarization (PHIP) is based on the chemical activation of parahydrogen,^[3]^ the nuclear spin‐0 isomer of H_2_. The hyperpolarization is produced upon chemical addition of parahydrogen to a substrate due to breaking the magnetic equivalence of protons originating from parahydrogen.^[4]^ The pair of H atoms must not be separated completely to observe the hyperpolarization, that is, the atoms must end up in the same molecule. These prerequisites are fulfilled, for instance, if parahydrogen molecules are involved in the homogeneous catalytic hydrogenations mediated by metal complexes.[^3b^, ^5^] However, toxicity issues require efficient methods of catalyst separation from hyperpolarized products^[6]^ and can limit their applications, especially in in vivo studies. Heterogeneous catalysts based on immobilized metal complexes, metal nanoparticles and binary metal compounds have also been demonstrated to enable PHIP.^[7]^ The separation is easier in this case as compared to homogeneous catalysts but achieved hyperpolarization levels are typically quite modest.

Metal‐free homogeneous catalytic systems may provide essentially new alternatives for PHIP. Naturally, substances made of main biogenic elements can be more environmentally and biologically friendly as compared to their metal‐containing counterparts. The mechanisms of H_2_ activation, however, are significantly different for the metal and nonmetal extremes. Pioneered by Stephan et al.,^[8]^ activation of H_2_ by frustrated Lewis pairs (FLPs) attracts extraordinary attention. First observations of spin hyperpolarization effects with metal‐free systems were reported for ansa‐aminoboranes (AABs), the intramolecular N‐B FLPs of 2‐boryl‐benzylamine core.^[9]^ Later, aromatic triphosphabenzene^[10]^ and several novel 2‐aminophenyl‐boranes^[11]^ were demonstrated to activate parahydrogen, leading to hyperpolarization effects. ^15^N‐Labeled analogues of those AABs also showed spontaneous hyperpolarization of their ^15^N nuclei.^[12]^ Several metal‐free pnictogen biradicaloids were also demonstrated to be efficient in hyperpolarizing nuclear spins.^[13]^ In all these cases, hyperpolarization effects were observed exclusively for catalyst‐parahydrogen adducts formed at the initial H_2_ activation step [Cat‐H_2_ in Scheme 1, Eq. (1)]. No successful spin hyperpolarization of target molecules produced by transfer of parahydrogen‐originating H atoms to a substrate of interest [Scheme 1, Eq. (2)] using metal‐free catalysts has been reported so far. At the same time, some FLP‐based systems are known to facilitate the full catalytic hydrogenation cycle for various unsaturated substrates.^[14]^ Finding such systems for parahydrogen would significantly broaden the scope of possible molecules that can be hyperpolarized using metal‐free PHIP.

**Scheme 1:**
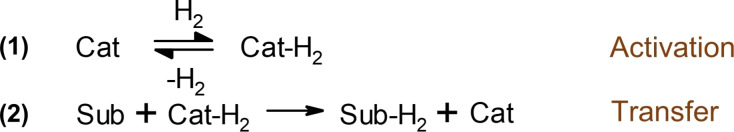
Steps of a substrate (Sub) hydrogenation over a catalyst (Cat).

Herein, we show that the metal‐free hydroborane catalyst HCAT^[14f]^ (Scheme 2) can provide hyperpolarization of various akenes in alkyne hydrogenations with para‐H_2_. Due to an unusual generally non‐pairwise mechanism of this process, only one of the double‐bond protons in the alkene products gains strong hyperpolarization. PHIP enables detecting ^1^H, ^11^B, and ^15^N NMR signals of the key catalytic cycle intermediates that were not accessible with the thermal polarization.

**Scheme 2:**
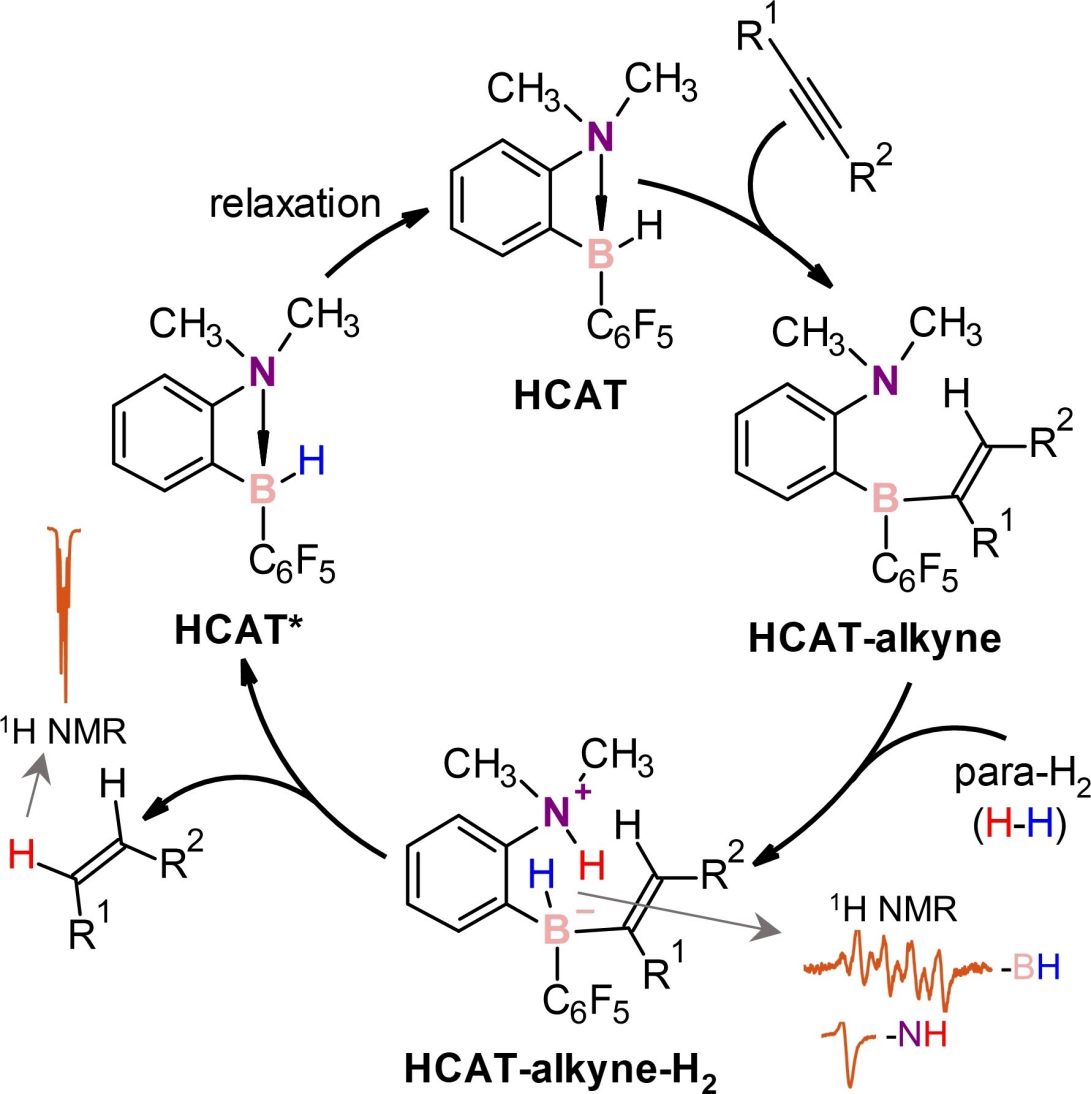
Mechanism of alkyne hydrogenation with para‐H_2_ over HCAT.

Several alkyne substrates were used to study the hydrogenation with parahydrogen (Table 1). The reaction was performed at room temperature under 6 bar of 92 % parahydrogen‐enriched H_2_ referred in the text simply as para‐H_2_. The NMR spectra were acquired after introducing para‐H_2_ into substrate‐HCAT solutions, examining the reaction in the high magnetic field (9.4 T, PASADENA‐type experiment,^[3b]^ see the Supporting Information).

**Table 1:** Substrates and reaction products in the studied hydrogenations.

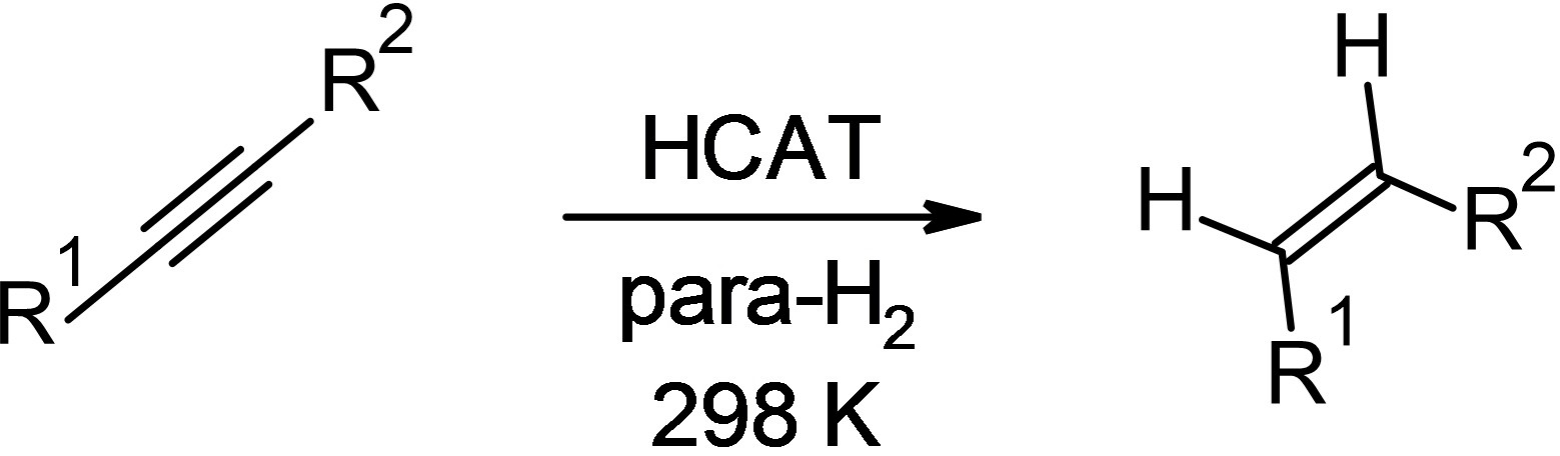			
Reaction	Substrate/structure		Product/structure
**R1**	**1**	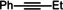	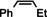
**R2**	**2**	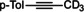	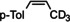
**R3**	**3**	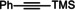	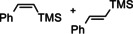
**R4**	**4**	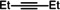	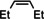
**R5**	**5**	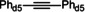	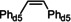

*p*‐Tol=*para*‐tolyl; TMS=trimethylsilyl; Ph_d5_=−C_6_D_5_.

Alkyne structures had an influence on the observed hyperpolarization effects, but general features were similar for all the substrates. For instance, the hydrogenation with para‐H_2_ of unsymmetrical 1‐ethyl‐2‐phenylacetylene (**1**) gave the ^1^H NMR spectrum shown in Figure 1 (upper trace). It reveals hyperpolarization through several antiphase and in‐phase signals. The antiphase signals correspond to the activated para‐H_2_ bound in the key HCAT‐alkyne‐H_2_ intermediates, in accord to the catalytic cycle in Scheme 2. Namely, NH and BH group hydrogens gained hyperpolarization after activation of para‐H_2_ molecules by the Lewis basic (N) and Lewis acidic (B) centers of the HCAT‐alkyne FLP species. The NH proton signals showed up as antiphase doublets, whereas the BH hydride signals appear as sets of four antiphase doublets due to the splitting induced by the coupling to the spin‐3/2 ^11^B nuclei. In fact, two sets of antiphase NH/BH ^1^H NMR signals corresponding to the different substrate arrangements were detected, as the initial addition of **1** to HCAT was not regioselective. The signal amplitudes for the two isomeric HCAT‐alkyne‐H_2_ intermediates are different (see, e. g., NH in Figure 1). The species with phenyl group adjacent to the boryl center (HCAT‐1b‐H_2_) were formed in a smaller concentration compared to its ethyl counterpart (HCAT‐1a‐H_2_), which is expectable based on the group steric bulkiness considerations. Noteworthy, these important intermediates were impossible to detect without the substantial signal enhancement provided by para‐H_2_, and their observation in spectra has never been reported before.

**Figure 1:**
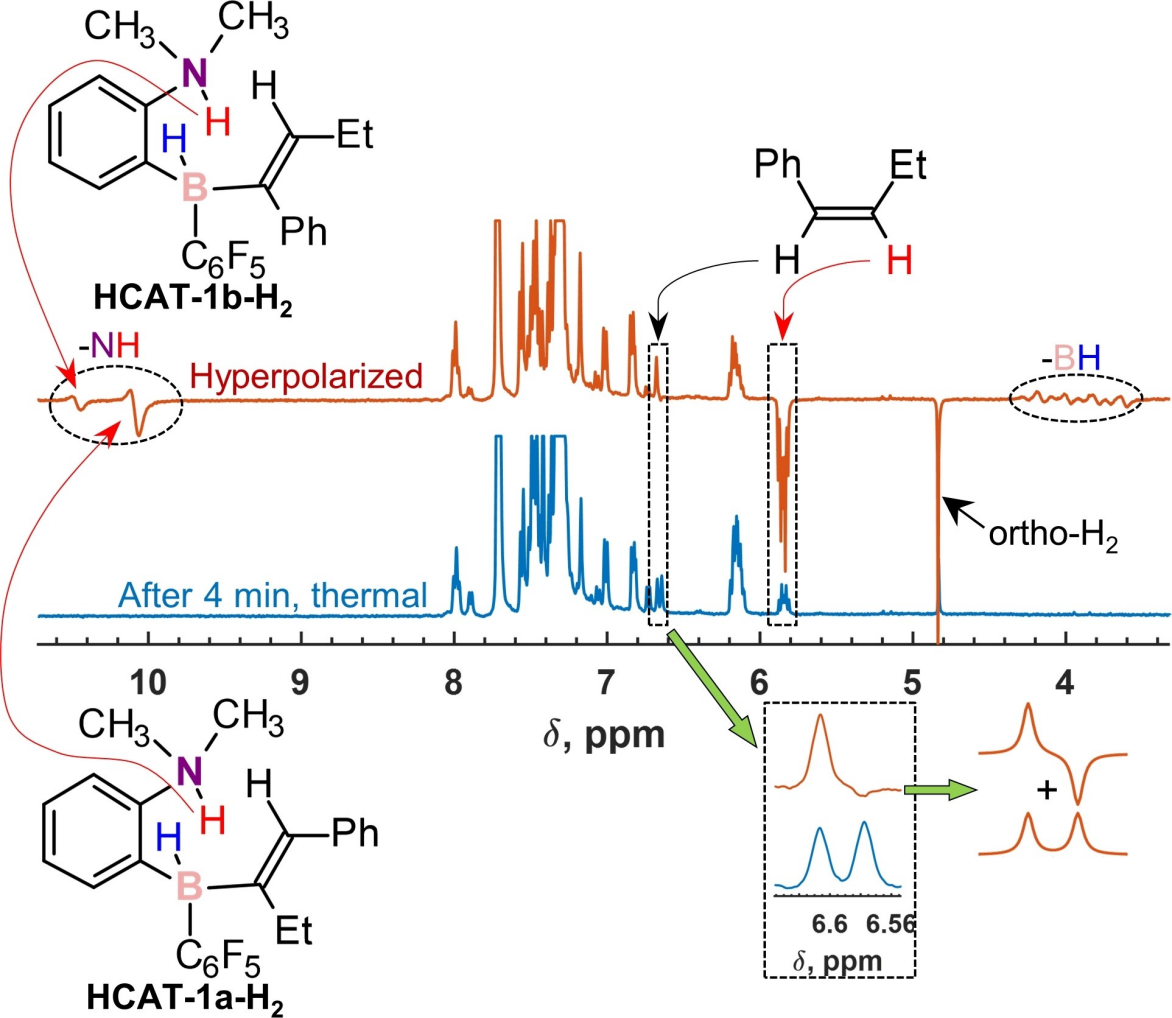
^1^H NMR spectra of HCAT‐catalyzed hydrogenation of 1‐ethyl‐2‐phenylacetylene (**1**) with para‐H_2_ (**R1**). The upper (red) spectrum was acquired by using a π/4 pulse just after introducing para‐H_2_ into a solution containing ca. 0.1 M HCAT and 0.3 M of **1** in toluene‐d_8_ at 298 K. The enhanced antiphase signals correspond to NH and BH group hydrogens of the two regioisomeric HCAT‐alkyne‐H_2_ intermediates (a and b). The enhanced in‐phase negative signals correspond to one of the double‐bond hydrogens in (*Z*)‐1‐phenylbut‐1‐ene product and ortho‐H_2_. The other double‐bond hydrogen gains a weak antiphase polarization (see the inset). The lower (blue) trace shows the thermal signal after 4 min.

The in‐phase signals correspond to the hyperpolarized reaction product, *ci*s‐alkene ((*Z*)‐1‐phenylbut‐1‐ene), and hyperpolarized ortho‐H_2_. The former observation is unusual, since one of the protons acquires strong net polarization (Figure 1, red hydrogen), whereas the other one does not. Only a negligible antiphase character was present in the signal of the other hydrogen (see the inset in Figure 1). Such an effect can be rationalized by considering the full catalytic cycle of HCAT (Scheme 2). This mechanism is not pairwise as, following the initial addition of para‐H_2_ to the HCAT‐alkyne adduct, elimination of the alkene product from HCAT‐alkyne‐H_2_ leads to the separation of the H pair. One H atom from para‐H_2_ (red) ends up in the alkene and another one (blue) remains in the regenerated HCAT*. The classical PASADENA[^3b^, ^4^] effect is not possible in these circumstances, simply because the intermolecular nuclear spin correlation inherited from para‐H_2_ cannot be transformed into the observable hyperpolarization. Therefore, we should not observe enhanced signals for the produced alkene. However, if the initial two‐spin order inherited from para‐H_2_ (∝ IHNz .IHBz ) can be transformed into the single‐spin net polarization (∝ IHNz ) at the stage of HCAT‐alkyne‐H_2_ intermediate, the transferred proton (red) will be hyperpolarized after the elimination of alkene (Scheme 2). Such a transformation can be driven by the spin relaxation transitions and was observed for metal complexes (Ru,Os)^[15]^ and also AABs.[^11^, ^12^] Simply speaking the proton at the N center of HCAT‐alkyne‐H_2_ gains a significant level of negative net polarization that is transferred to the alkene product. Moreover, the same phenomenon can lead to the negative polarization of ortho‐H_2_, as H_2_ addition to HCAT‐alkyne adduct is a reversible process that can release the hyperpolarized ortho‐H_2_. We note that there are few examples of one‐hydrogen polarization reported with metal complex catalysts in hydroformylation reactions^[16]^ and exchange of water ligands^[17]^ as well as with metal nanoparticles for exchanging water,^[18]^ but the catalyst nature, reactions and mechanisms are essentially different. In addition to the relaxation‐based mechanism, coherent mixing was used to explain the origin of single‐spin net polarization in there. This origin can be ruled out in our case because it should lead to the positive net polarization of alkenes (see the Supporting Information for details), whereas we observe the negative one.

Features like the ones observed in **R1** were also present in hydrogenations of the other alkynes (Table 1). In the case of unsymmetrical alkynes **R1** and **R2** (Figures 1 and 2), two regioisomeric HCAT‐alkyne adducts were formed. Both isomers reacted with para‐H_2_, resulting in two sets of antiphase signals corresponding to two HCAT‐alkyne‐H_2_ intermediates. However, the rates of alkene elimination from these intermediates were noticeably different, leading to the dominating negative net hyperpolarization of only one proton at the double bond of the products, the proton that had aliphatic substituent in the geminal position. Interestingly, the reaction with unsymmetrical TMS substrate **3** revealed hyperpolarization of stereoisomeric *cis* and *trans* HCAT‐alkyne‐H_2_ intermediates instead of regioisomers (Figure 2, **R3**). We observed higher product formation rates from the *cis*‐derivatives, leading to the much faster accumulation of *cis*‐alkene in the reaction mixture. Moreover, this product revealed hyperpolarization, whereas *trans* alkene did not. In the latter case, likely the slow product accumulation does not allow observing any effects before the hyperpolarization is destroyed by the nuclear spin relaxation. The mechanism of *cis*–*trans* isomerization mediated by the stabilizing β‐effect of silicon is discussed for **R3** in the Supporting Information (Scheme S4).

**Figure 2:**
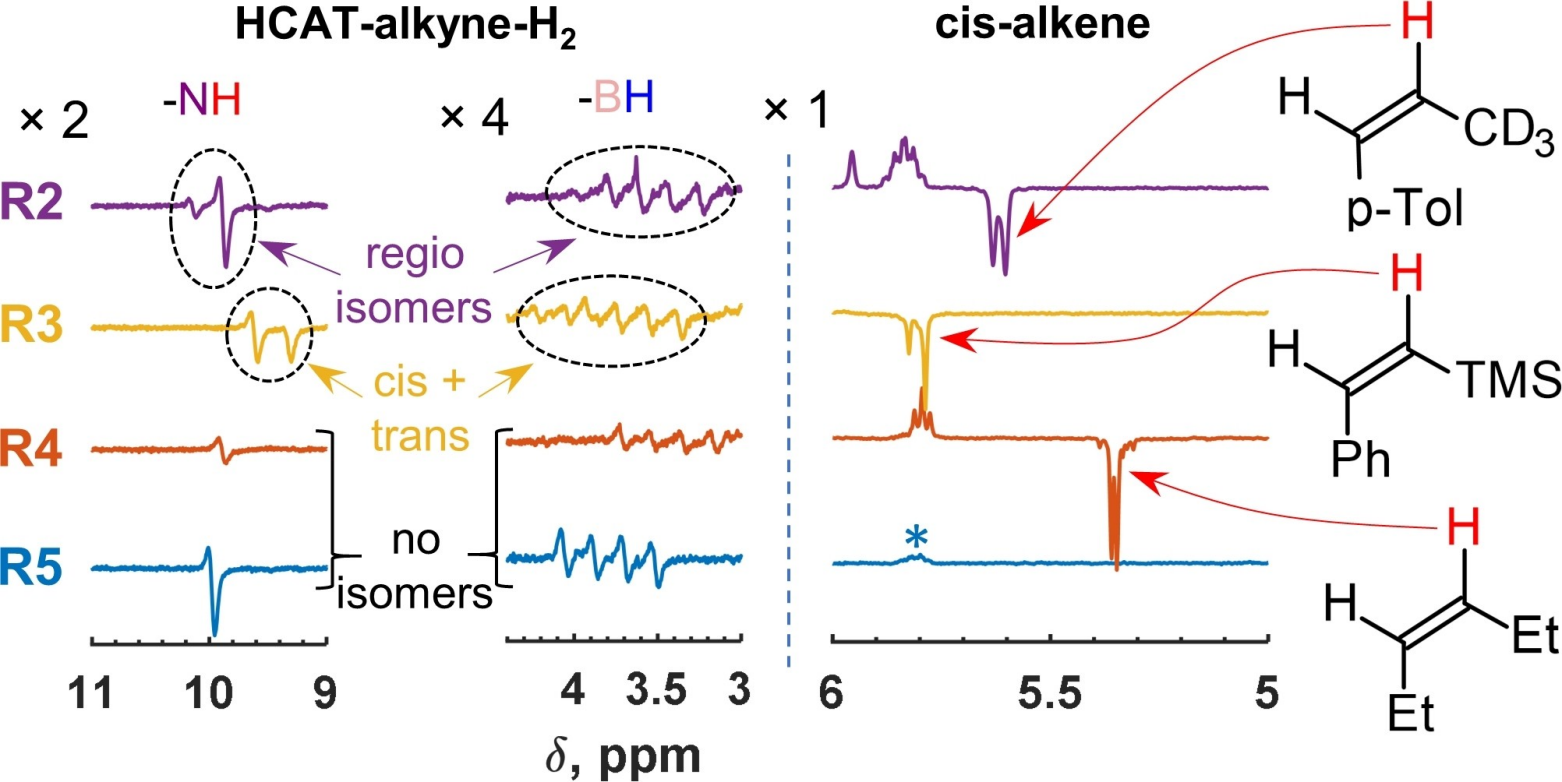
Selected fragments of ^1^H NMR spectra showing hyperpolarized species produced in the course of hydrogenation reactions **R2**–**R5** over HCAT catalyst at 298 K in toluene‐d_8_. All spectra were measured immediately after para‐H_2_ was introduced into the corresponding substrate‐HCAT solutions. Left: the signals of NH and BH hydrogens of HCAT‐alkyne‐H_2_ intermediates; right: the signals of the hyperpolarized protons in *cis*‐alkenes. The signal of the double‐bond protons of (*Z*)‐1,2‐diphenylethylene‐d_10_ is marked with an asterisk. The spectral regions are scaled by different factors indicated in the figure.

In the case of symmetrical substrates (**R4** and **R5**), single sets of the antiphase signals for NH and BH groups of HCAT‐alkyne‐H_2_ intermediates were detected (Figure 2, two bottom traces). Due to the molecular symmetry of **4** and **5**, only one HCAT‐alkyne regioisomer could be formed, while *cis*–*trans* isomerization did not take place under employed experimental conditions. Moreover, solely hex‐3‐yne hydrogenation (**R4**) with para‐H_2_ resulted in hyperpolarization of the double‐bond protons of the corresponding *cis*‐alkene. Due to the symmetry of the product, it is unclear whether only one of the protons was hyperpolarized. However, taking into account the results with unsymmetrical molecules, we can claim that it is most likely the case. The reaction of perdeuterated tolane (**5**) was very slow due to the low rate of the corresponding alkene elimination from the HCAT‐alkyne‐H_2_ intermediate, preventing observation of the hyperpolarized diphenylethylene‐d_10_.

In addition to ^1^H NMR, all the hydrogenation reactions (**R1**–**R5**) allowed observing spontaneous ^11^B hyperpolarization for HCAT‐alkyne‐H_2_ intermediates (Figures S3–S17). For instance, ^11^B NMR spectra obtained for **R1** reveal antiphase signals of these species immediately after introducing a fresh potion of para‐H_2_ (Figure 3a). The signals disappear in 1 min time as a result of para‐H_2_ conversion into normal H_2_. Experiments with normal H_2_ did not lead to the observation of these intermediates neither in ^11^B nor in ^1^H NMR, implying a substantial signal enhancement (>100 for **1**). Data for the other reactions are shown in Figures S6–S17.

**Figure 3:**
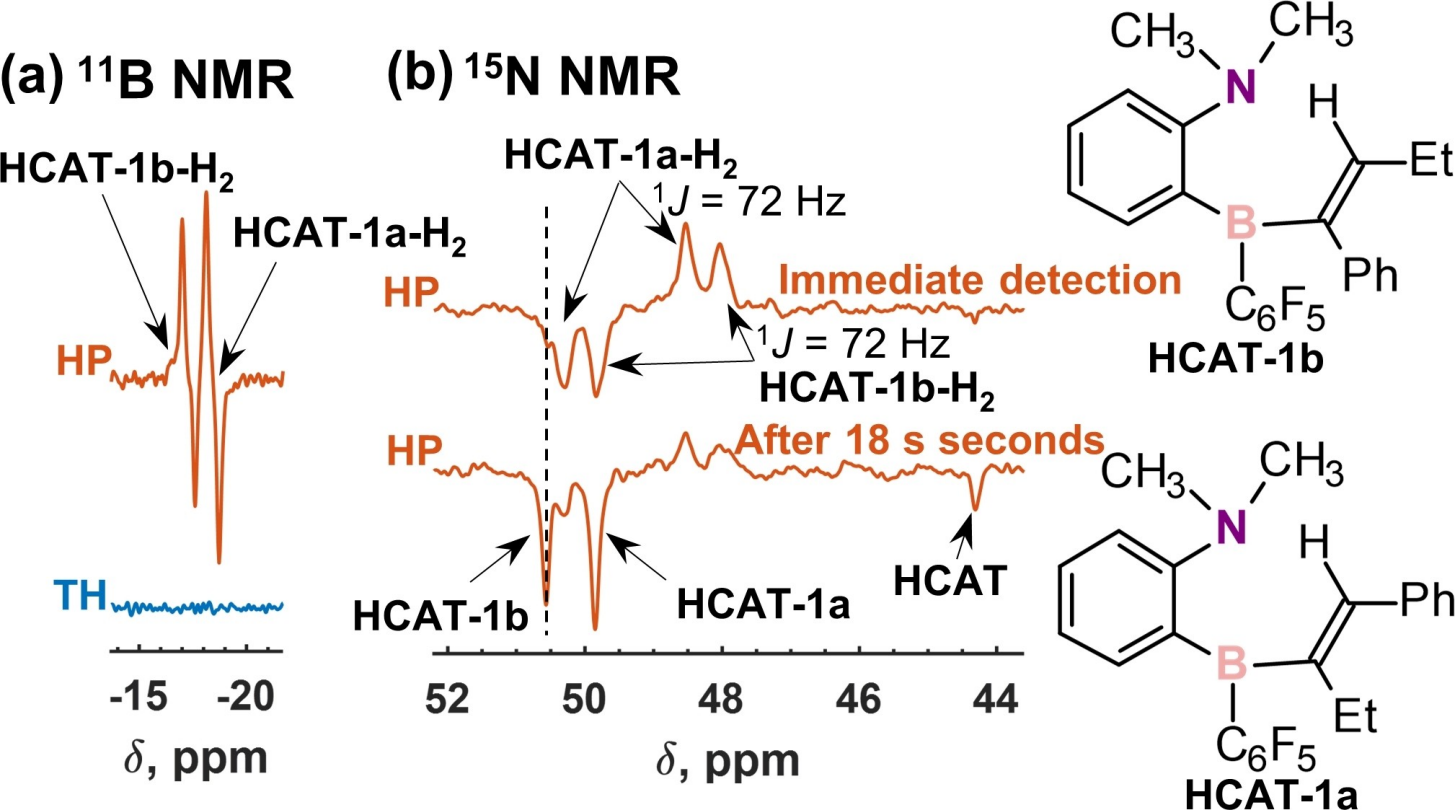
a) ^11^B NMR spectra measured after adding para‐H_2_ to a solution of HCAT (0.1 M) and **1** (0.3 M) in toluene‐d_8_ at 298 K (red trace) and after para‐H_2_ was converted into normal H_2_ (blue trace). b) ^15^N NMR spectra acquired immediately after introducing a fresh portion of para‐H_2_ (upper trace) and 18 s later (lower trace) to ^15^N‐HCAT (0.1 M) and **1** (0.3 M) in toluene‐d_8_ at 298 K. The signals of the hyperpolarized species are marked using arrows in the spectra. See Figures 1 and 3 for their structures. HP=hyperpolarized, TH=thermal.

Finally, we probed the influence of ^15^N‐labeling of HCAT. The corresponding compound was synthesized as described in Scheme S2. It was found that labelling has only a moderate effect on the ^1^H hyperpolarization levels (Figure S3). Within the accuracy of our measurements, in either cases, ^1^H signal enhancements were of two orders of magnitude (Tables S1–S3). However, it opened a way to detect ^15^N NMR of the HCAT‐alkyne‐H_2_ intermediates in the studied reactions, since the use of para‐H_2_ led to the spontaneous ^15^N hyperpolarization in the similar way as it was for ^11^B. Figure 3b shows ^15^N NMR spectra acquired using ^15^N‐HCAT in **R1**. One can see that two antiphase doublets (^1^ *J*_HN_≈72 Hz) corresponding to HCAT‐1a‐H_2_ and HCAT‐1b‐H_2_ show up immediately after introducing a fresh portion of para‐H_2_. Interestingly, waiting for several seconds after that leads to the appearance of in‐phase negative signals of HCAT‐1a, HCAT‐1b and HCAT. Apparently, the hyperpolarization of these species is a consequence of accumulation of the negative net ^15^N polarization in the HCAT‐alkyne‐H_2_ intermediates and its further spreading by reversible exchange processes within the catalytic cycle. This is a related effect to that of the hyperpolarization of free ligands in signal amplification by reversible exchange (SABRE).^[19]^ Metal‐free SABRE was reported in the para‐H_2_ activations with ansa‐aminoboranes^[12]^ and pnictogen biradicaloids,^[13a]^ but this is the first time when it is reported in metal‐free hydrogenation reactions. Without the use of para‐H_2_, a 32‐scan accumulation could lead to the detection of HCAT‐1a and HCAT‐1b in ^15^N NMR, whereas HCAT‐1a‐H_2_, HCAT‐1b‐H_2_ and HCAT were still not visible. This observation shows the advantage of using hyperpolarization for the rapid detection of scarcely populated intermediates by the multinuclear NMR. Similar data for the other reactions are presented in Figures S3–S17. Altogether, PHIP allowed to collect a large set of the multinuclear NMR data (^1^H,^11^B,^15^N) characterizing these intermediates. Discussion of the polarization transfer mechanisms leading to the spontaneous ^11^B and ^15^N hyperpolarization is beyond the scope of this Communication. Most likely it is facilitated by the relaxation‐driven transitions that can connect the two‐spin longitudinal magnetization mode inherited from para‐H_2_ with the heteronuclear two and single‐spin modes as discussed, for instance, in ref. [12] and papers cited therein.

In summary, for the first time metal‐free catalytic hydrogenation with para‐H_2_ was shown to provide hyperpolarized products. ^1^H NMR signal enhancements of two orders of magnitude were obtained for alkenes at 9.4 T (Table S1) in the hydrogenations of alkynes **1**–**4** when HCAT was used as catalyst. Predominantly only one of the two incorporated hydrogens at the double bond of the products was hyperpolarized. This process is mediated by a non‐pairwise hydrogenation mechanism accompanied by accumulation of the negative net hyperpolarization at the NH proton site in the HCAT‐alkyne‐H_2_ intermediates. We observed effects of regio‐ and stereoselectivity of alkyne addition to HCAT and a difference in reaction rate of the alkene elimination from these important intermediates. Moreover, HCAT‐alkyne‐H_2_ species were characterized by hyperpolarized ^1^H, ^11^B and ^15^N NMR spectroscopy. Multinuclear NMR spectra were not accessible when using thermal polarization (Figures S3–S17, Tables S2 and S3). This is only the first study of metal‐free hydrogenation of alkynes with para‐H_2_; we envision higher activities, reaction rates and signal enhancements to be achieved by optimizing conditions and the structure of HCAT catalyst as well as by in‐depth studies of spin dynamics leading to the net hyperpolarization at NH site. We believe that the intramolecular nature of HCAT plays a key role for the hyperpolarization, as our preliminary experiments with intermolecular FLPs did not reveal any hyperpolarization effects. Further work towards designing ansa‐systems as a broad class of metal‐free catalysts for PHIP and SABRE is ongoing.

## Conflict of interest

The authors declare no conflict of interest.

## Supporting information

As a service to our authors and readers, this journal provides supporting information supplied by the authors. Such materials are peer reviewed and may be re‐organized for online delivery, but are not copy‐edited or typeset. Technical support issues arising from supporting information (other than missing files) should be addressed to the authors.

Supporting Information
