# Supplementary material for: Parahydrogen‐Induced Polarization in Hydrogenation Reactions Mediated by a Metal‐Free Catalyst
Source: Chemistry. 2022 Jan 19;28(8):e202103501. doi: 10.1002/chem.202103501 (PMC9303582; doi:10.1002/chem.202103501)
Supplement: Supplementary file 1 — Supporting Information [file CHEM-28-0-s001.pdf]

Chemistry–A European Journal

Supporting Information

Parahydrogen-Induced Polarization in Hydrogenation Reactions Mediated by a Metal-Free Catalyst

Danila O. Zakharov, Konstantin Chernichenko, Kristina Sorochkina, Shengjun Yang, Ville-Veikko Telkki, Timo Repo, and Vladimir V. Zhivonitko*

– CONTENTS –

1 General information....................3

1.1 Instruments and chemicals....................3

1.2 Synthesis of ^{15}N-PrHCAT....................4

1.3 Para-H_2 experiments....................6

2 Hyperpolarization of alkene products....................7

2.1 ^{1}H NMR spectra obtained using natural (non-labelled) HCAT....................9

2.2 ^{1}H NMR spectra obtained using ^{15}N-labelled HCAT....................10

2.3 ^{1}H NMR signal enhancements of alkene products....................11

3 Characterization of intermediates using PHIP....................12

3.1 ^{1}H, ^{11}B and ^{15}N NMR spectra of reaction intermediates....................12
3.1.1 Hydrogenation of 1....................12
3.1.2 Hydrogenation of 2....................15
3.1.3 Hydrogenation of 3....................18
3.1.4 Hydrogenation of 4....................21
3.1.5 Hydrogenation of 5....................24

3.2 Signal enhancements for HCAT-alkyne-H_2 intermediates and HCAT-alkyne adducts....26

4 Ruling out coherent mixing as a mechanism of one-hydrogen hyperpolarization in HCAT catalyzed hydrogenations....................27

5 Stereoisomerization in HCAT catalyzed hydrogenation of 3....................29

6 References....................32

1 General information

1.1 Instruments and chemicals

Multinuclear NMR spectra were acquired on a 400 MHz Bruker AV 400 NMR spectrometer equipped with a broad-band 5 mm radiofrequency probe. All spectra were recorded at 25°C. Parahydrogen-enriched H_2 gas (92%) referred to in the main text as simply para-H_2 was produced using a Bruker parahydrogen generator.

Non-labelled HCAT catalyst, N,N-dimethyl-2-[(pentafluorophenyl)boryl]aniline, was prepared from its precursor, N,N-dimethyl-2-[bis(pentafluorophenyl)boryl]aniline (PrHCAT), by keeping its toluene solution under 6 bar pressure of H_2 at 80 °C overnight.[S1]

Scheme S1. Generating HCAT under H_2 atmosphere from PrHCAT

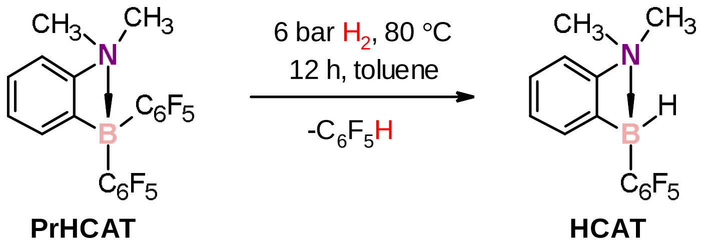

Typically, this procedure was done *in situ* in gas-tight NMR tubes. In selected experiments, we also isolated HCAT as a light green powder by evaporating the solvent *in vacuo*. The resulting material was used to prepare samples without further purification.

PrHCAT was synthesized via synthetic procedures reported elsewhere.[S2] The ^{15}N-labelled counterpart of PrHCAT, ^{15}N-PrHCAT, was synthesized according to the method described in the next section. Alkyne substrates **1-5** and reagents to perform synthesis of ^{15}N-labeled HCAT were obtained from commercial sources (**1-4** Sigma-Aldrich and **5** Cambridge Isotope Labs). Sample preparations were performed under inert (Ar) atmosphere to avoid possible side reactions. Deuterated toluene-d8 was obtained from Eurisotope and additionally dried by keeping it over molecular sieves (3 Å).

1.2 Synthesis of ^{15}N-PrHCAT

^{15}N-labelled precursor of HCAT (^{15}N-PrHCAT) was synthesized according to following procedure. Scheme S2 shows the general synthetic route.

Scheme S2. General scheme of synthesis of ^{15}N-PrHCAT

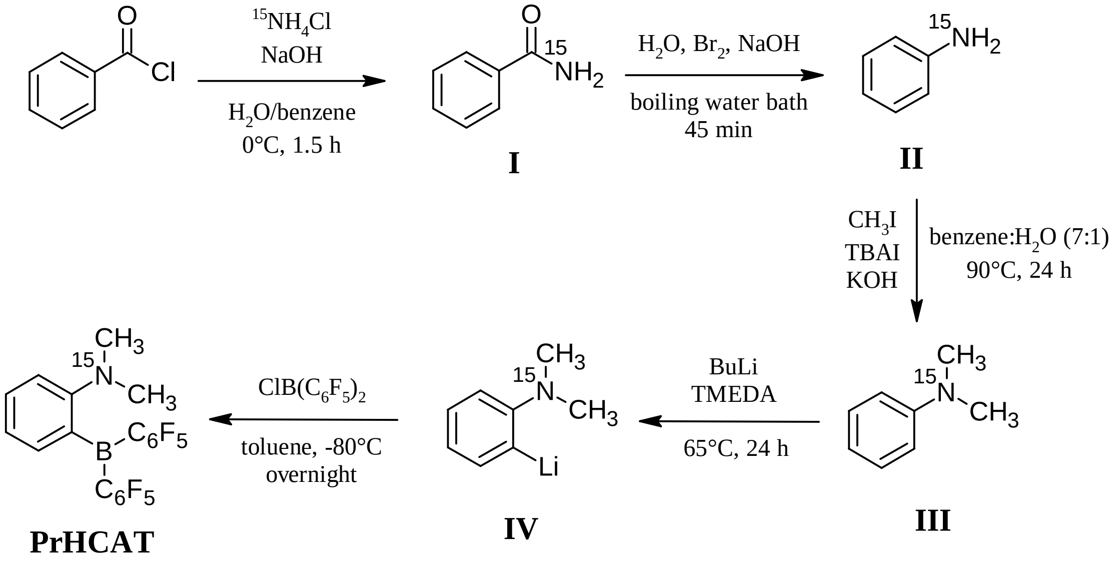

^{15}N-Benzamide (**I**).[S3] To the ice cooled solution of $^{15}NH_4Cl$ (5.0 g, 91.8 mmol) in distilled water (46 mL) in a 1 L round-bottom flask equipped with a magnetic bar, benzene (10 mL) was added to cover the aqueous layer. An ice cooled solution of 7.75g (193.7 mmol) of NaOH in water (35 mL) was added to the aqueous layer through a pipette. A solution of benzoyl chloride (10.6 mL, 91.6 mmol) in 345 mL of benzene was poured rapidly and sealed tightly with stopper. The resulting solution was stirred at room temperature. After 1.5 hours, the solution was cooled in ice bath to produce more precipitate and then was filtered through a Büchner funnel. The filter cake was washed with ice-cooled benzene (35 mL × 2) and squeezed to dry. The filtrate was separated using separatory funnel. The organic phase was evaporated under reduced pressure to give a concentrated solution. The aqueous layer was extracted by $CHCl_3$ in small portions (10 mL × 6). The extracts were combined and dried over anhydrous Na_2SO_4. After filtration, the solvent was evaporated under reduced pressure to give another concentrated solution. The two concentrated solutions were combined and crystallized to give the resulting ^{15}N-benzamide. All the products were combined and then dried in an oven at 85-90 °C overnight. Totally, 10.39 g (85 mmol) product was obtained, yield: 92.7 %.

^{15}N NMR (toluene-d8): δ ppm 96.15 (s).

^{15}N-Aniline (**II**).[S3] To an ice cooled NaOH (5.0 g, 125 mmol) solution in distilled water (47 mL) in 100 mL round-bottom flask, Br_2 (2.75 mL, 53.7 mmol) was slowly added in 7 minutes with

vigorous stirring. Finely grounded ^{15}N-benzamide (5.0 g, 41.3 mmol) was added to the solution in small portions over 3 minutes. The suspension was stirred for another 20~30 minutes, then transferred to another round-bottom flask. Another solution of NaOH (2.85 g, 71.2 mmol) in distilled water (9.0 mL) was added to the suspension and heated in boiling water bath. After 40~50 minutes, the refluxing condenser was replaced with a steam distillation setup and heating of the steam generator was started. Steam distillation was continued till no oily phase distillate was coming out. After cooling, the residue in the reaction flask was acidified with 5% hydrochloric acid and heated at boiling for 15 min. The solution was neutralized with NaOH solution and the steam distillation run again. The distillates were combined, extracted with diethyl ether, and dried using solid NaOH. After filtration, the solid was filtered off and the filtrate was concentrated under reduced pressure to give the yellowish oil. Totally, 3.04 g product was obtained, yield: 79%.

^{15}N NMR (toluene-d8): δ ppm 55.06 (s).

^{15}N,^{15}N-Dimethylaniline (**III**).[S4] To a solution of ^{15}N-aniline (2.0 g, 21.48 mmol) in benzene (28 mL) and distilled water (4 mL) in 100 mL round-bottom flask, TBAI (0.56 g, 1.5 mmol) and KOH (3.01 g, 53.7 mmol) were charged. CH_3I (3.4 mL, 53.7 mmol) was then added dropwise to the mixture at room temperature. The resulting solution was heated at 90 °C and kept at this temperature for 24 hours. After cooling down to room temperature, the mixture was extracted with diethyl ether (20 mL × 3). The organic layer was combined and washed with water (15 mL × 3), saturated $NaHCO_3$ solution (15 mL × 3) and dried with anhydrous Na_2SO_4. After filtration, the solution was concentrated under reduced pressure. The residue was subjected to column chromatography with silica gel 60 (eluent: hexane:ethyl acetate = from 80:1 to 10:1), giving (1.32 g) yellowish liquid in yield of 51%.

^{15}N NMR (toluene-d8): δ ppm 44.86 (s).

2-(^{15}N,^{15}N-Dimethylamino)phenyllithium (**IV**).[S5] A solution of ^{15}N,^{15}N-Dimethylaniline (1.046 mL, 8.25 mmol) and TMEDA (0.185 mL, 1.24 mmol) was degassed in Schlenk flask and kept under argon. A 1.6 M solution of n-buthyllithium (5.156 mL, 8.25 mmol) was added to the reaction mixture in small portions using syringe. The resulting solution was heated at 65 °C for 24 hours under argon atmosphere. A large amount of white precipitate was formed after heating. Hexane (15 mL) was added after cooling down to room temperature and solution was stirred for 5~10 minutes. After that the liquid was separated from the solid by decantation. The residue was washed with hexane (15 mL) and formed liquid phase was removed again by decantation. Resulting white solid was dried in Schlenk flask *in vacuo*. Totally, 0.615 g of the product was obtained, yield: 59%.

^{15}N NMR (toluene-d8): δ ppm 33.85 (s).

2-[bis(pentafluorophenyl)boryl]-^{15}N,^{15}N-dimethylaniline (**PrHCAT**).[S2] To a solution of chloro[bis(pentafluorophenyl)]borane (1.796 g, 4.72 mmol) in 10 mL of toluene at -80 °C a suspension of 2-(^{15}N,N-Dimethylamino)phenyllithium (0.600 g, 4.72 mmol) in 5 mL of toluene was added in one portion via canula. Organolithium was additionally rinsed with 5 mL of toluene and transferred into a Schlenk tube. The resulting reaction mixture was warmed to room temperature naturally and stirred overnight. After stirring, this solution was evaporated to a half of the volume and a 5 mL portion of hexane was added. After that the liquid phase was partially removed by decantation, and 15 mL of toluene were added to remaining suspension. The liquid was decanted, and grey precipitate was dried *in vacuo* to give 1.55 g of crude compound (reaction yield: 70%). 550 mg this material was recrystalized in 10 mL of hexane, filtered and 10 mL of toluene was added to Schlenk tube. Residue was filtered off and the solution was dried in vacuo. This purification procedure was repeated twice and 350 mg of white crystals were obtained. Recrystallization yield: 64%.
^{15}N NMR (toluene-d8): δ ppm 49.56 (s).

1.3 Para-H_2 experiments

NMR samples were prepared by making solutions of HCAT (0.05 mmol) in 0.420 mL of dry degassed toluene-d8 in 5 mm Wilmad gas-tight NMR tubes and adding 0.15 mmol of desired alkyne (**1**-**5**) under inert atmosphere. Prepared in this way, resulting mixtures contained HCAT-alkyne adducts, HCAT and free alkynes. At the next step, the sample tubes were gently charged with 6 bars of para-H_2, avoiding gas-liquid mixing. Thereafter, tubes were vigorously shaken for *ca.* 3 s just before insertion to the NMR magnet, placed inside spectrometer, and NMR spectra were recorded. A single tube shaking allowed measuring several NMR spectra (^{1}H, ^{11}B or ^{15}N) before para-H_2 completely converted into normal H_2 and the hyperpolarization completely decayed.

^{1}H NMR hyperpolarization effects observed using π/4-pulses always demonstrated antiphase character of signals in the case of HCAT-alkyne-H_2 intermediates, which was practically independent from whether measurement was performed immediately after insertion of samples to the magnet or after some time spent in the magnet. This indicated that in all cases we observed PASADENA[S6,7] type hyperpolarization, *i.e.*, predominantly the hyperpolarization of species that were generated inside the magnet. ALTADENA[S8] signal shapes (in-phase signals for the NH and BH protons having opposite phases) were not observed in any experiments, which is most likely due to the short T_1 relaxation of NH and BH protons in the intermediates (<1 s). It is significantly shorter than the time required for the sample insertion (*ca.* 3 s). Expectedly, the use of π/2-pulses

decreased the amplitude of antiphase NH and BH peaks. In contrast, the amplitude of enhanced signals of hyperpolarized alkene products increased when $\pi/2$-pulses were applied, and the signal shape did not change in this case, indicating the net polarization character of the hyperpolarization acquired by alkenes.

In the reported NMR experiments, spontaneous hyperpolarization of ^{11}B (see Figures 1 and 2 in the main text as well as Figures S4, S7, S10, S13 and S15 here) and ^{1}H hyperpolarization of HCAT-alkyne-H_2 intermediates (see Figures S1-S3, S6, S9, S12 and S14) showed fast build-up in the presence of para-H_2, which in principle allowed doing several scan accumulations of signals of the hyperpolarized species. In the case of spontaneous ^{15}N hyperpolarization, the distribution of hyperpolarized ^{15}N NMR signals was dependent on the time spent after adding para-H_2, showing stronger effects for HCAT-alkyne-H_2 intermediates in the first moments and appearance of signals from HCAT-alkyne adducts in the later moments (see Figure 3 in the main text as well as Figures S5, S8, S11, S14 and S17 here). This effect can be explained by the reversible exchange within the catalytic cycle that requires some time to transfer hyperpolarization from HCAT-alkyne-H_2 to HCAT-alkyne, as explained in the main text.

The following sections show NMR spectra, estimates of signal enhancements and mechanistic schemes supporting conclusions made in the main text.

2 Hyperpolarization of alkene products

In this section, we present structures of discussed compounds (Scheme S3), ^{1}H NMR spectra that demonstrate hyperpolarization effects for alkenes obtained as a result of hydrogenation over non-labelled (Figures S1) and ^{15}N-labelled (Figure S2) HCAT catalyst as well as a table with estimates of the signal enhancements of alkene signals provided by PHIP (Table S1).

Scheme S3. Structures of reaction products and intermediates discussed in the text. Hydrogens marked with “*” correspond to positions of hyperpolarized protons in the alkene products.

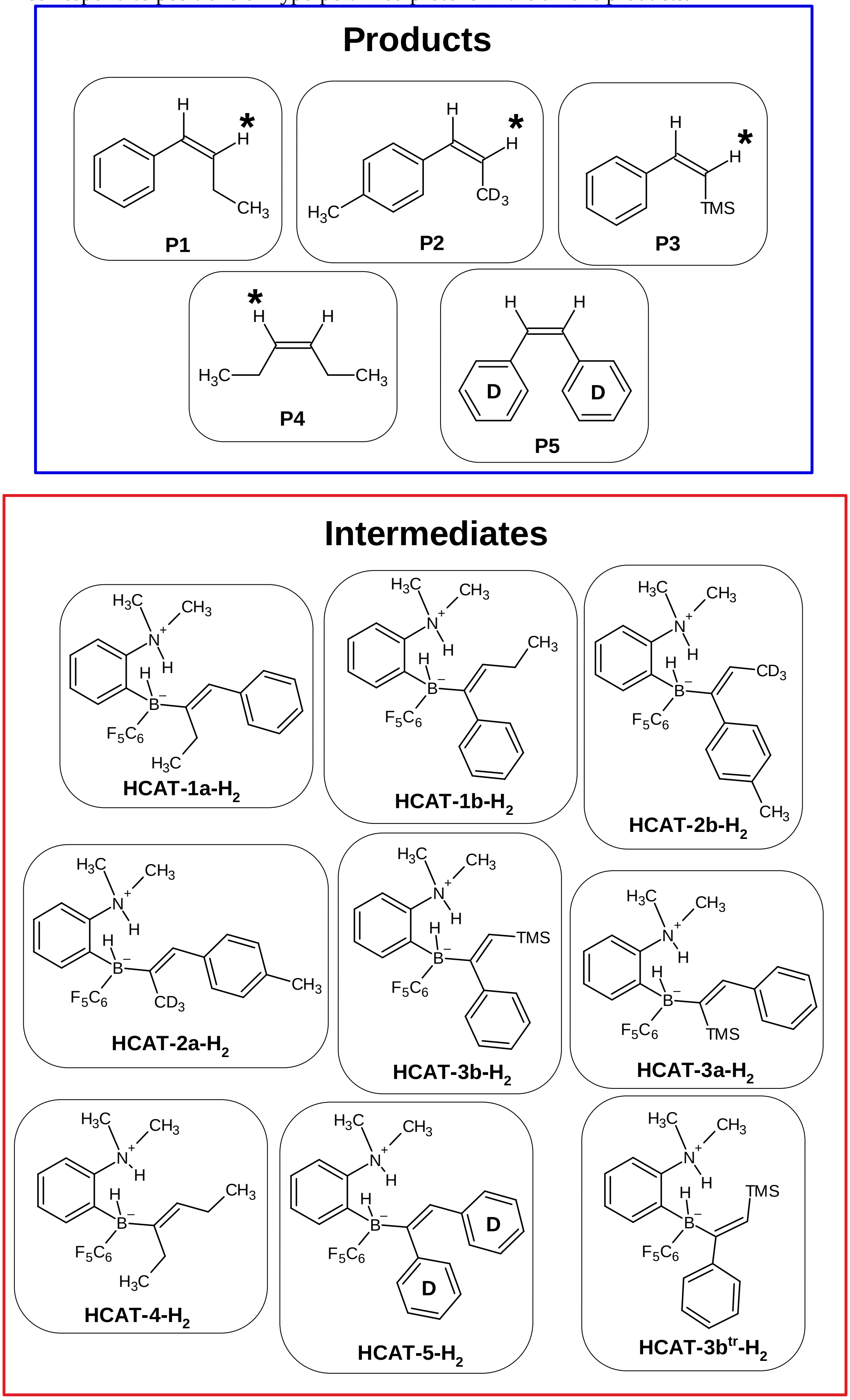

2.1 ^{1}H NMR spectra obtained using natural (non-labelled) HCAT

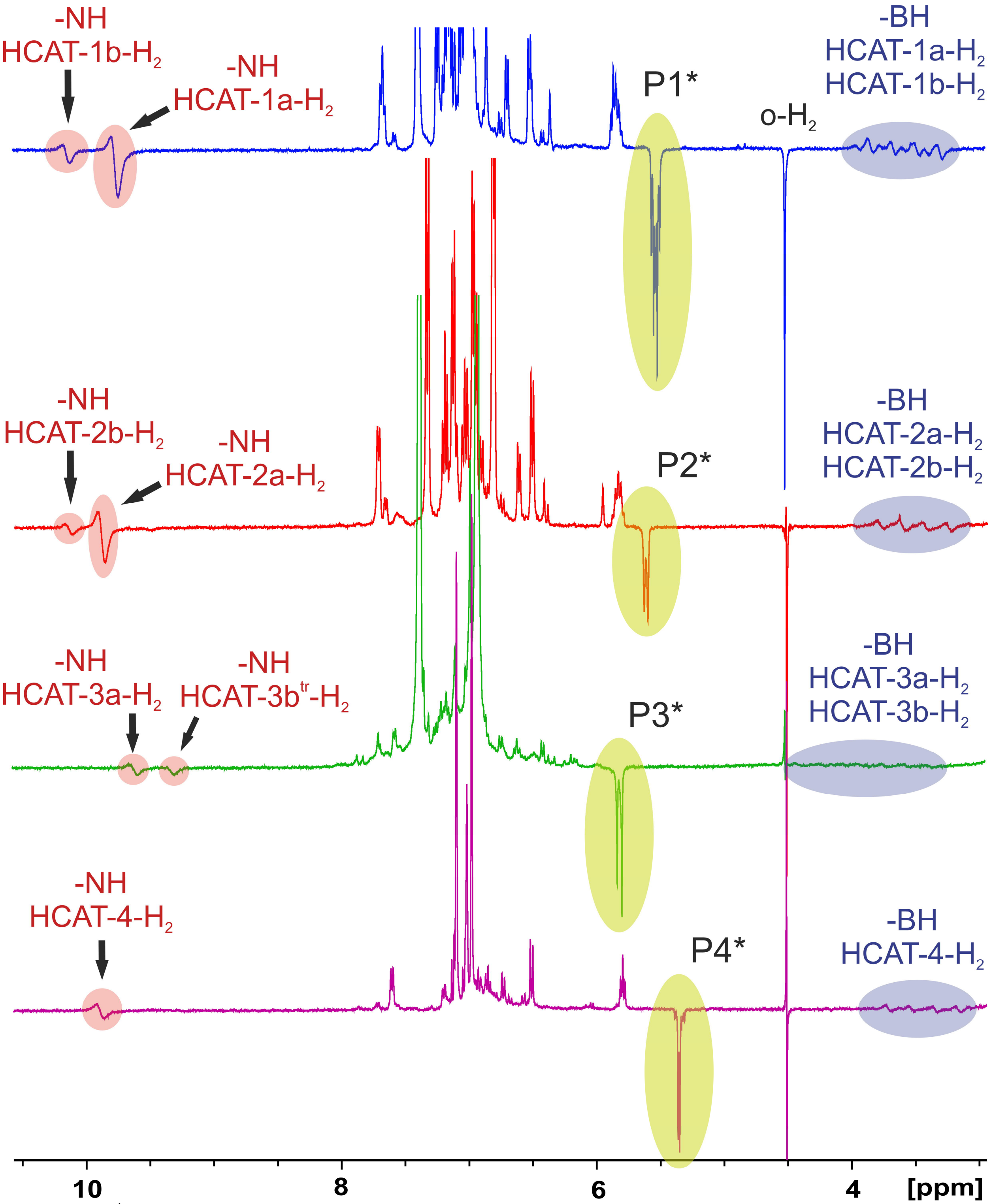

Figure S1. ^{1}H NMR spectra acquired using non-labelled HCAT in hydrogenations of alkynes **1-4** with para-H_2 showing hyperpolarization of alkene products **P1-P4**. Positions of hyperpolarized ^{1}H nuclei in the alkenes are marked with "*" in Scheme S3. Hyperpolarization effects are visible also for HCAT-alkyne-H_2 intermediates, -NH/-BH group ^{1}H NMR signals, and ortho-H_2. Positions of signals of interest are highlighted using transparent ovals. Some high-intensity signals corresponding to thermally polarized protons are off-scale and clipped in for a better visibility of other signals.

2.2 ^{1}H NMR spectra obtained using ^{15}N-labelled HCAT

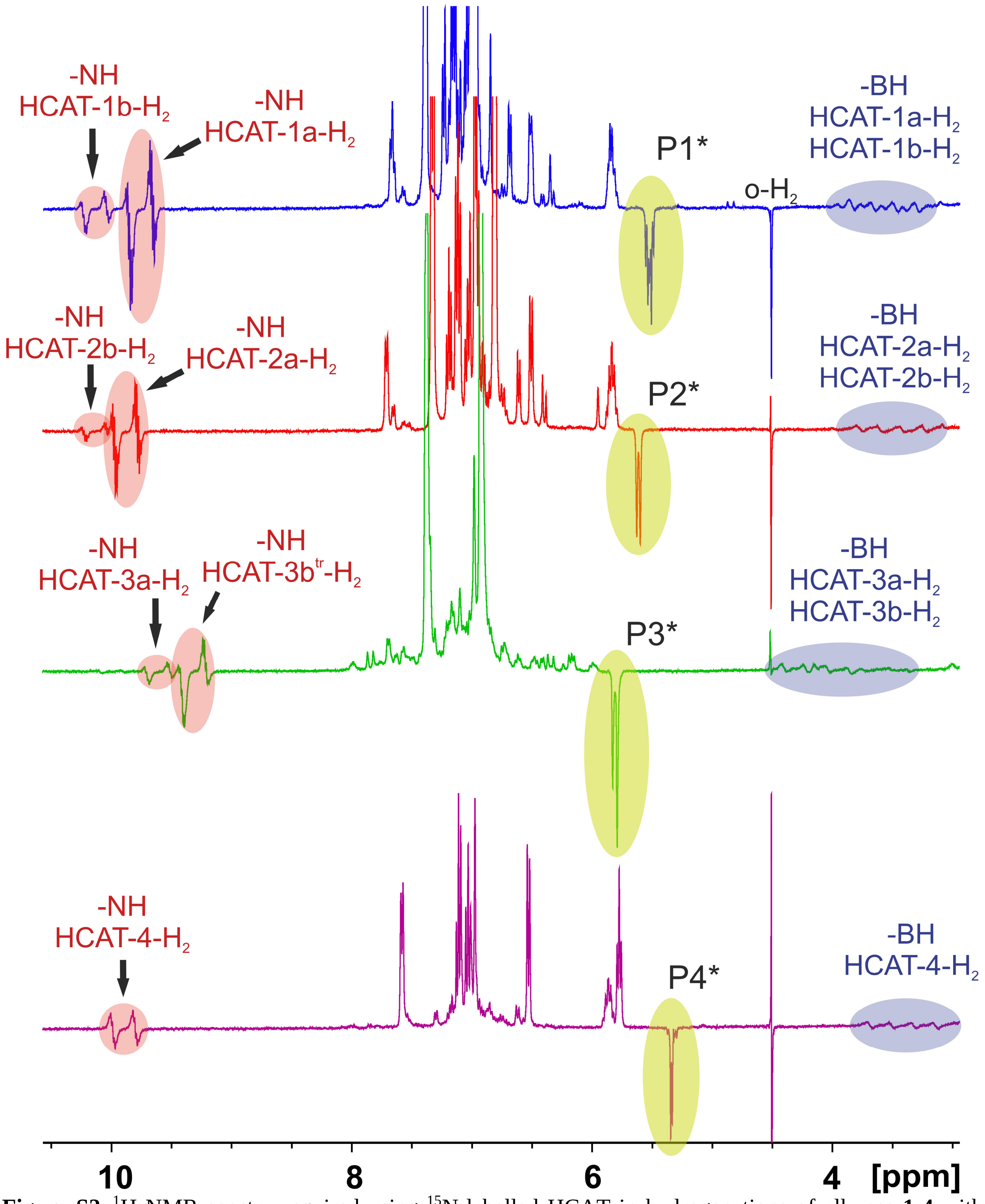

Figure S2. ^{1}H NMR spectra acquired using ^{15}N-labelled HCAT in hydrogenations of alkynes **1-4** with para-H_2 showing hyperpolarization of alkene products **P1-P4**. Positions of hyperpolarized ^{1}H nuclei in the alkenes are marked with "*" in Scheme S3. Hyperpolarization effects are visible also for HCAT-alkyne-H_2 intermediates, -NH/-BH group ^{1}H NMR signals, and ortho-H_2. Positions of signals of interest are highlighted using transparent ovals. Some high-intensity signals corresponding to thermally polarized protons are off-scale and clipped in for a better visibility of other signals.

2.3 ^{1}H NMR signal enhancements of alkene products

^{1}H NMR enhancement factors ($\varepsilon = A^{HP}/A^{TH}$) were estimated by determining the absolute ratios of signal amplitudes with (A^{HP}) and without (A^{TH}) hyperpolarization for the double-bond hydrogens that were hyperpolarized in our experiments. For the studied alkenes, these hydrogens are marked with "*" in Scheme S3.

As para-H_2 was present in the solution for a relatively long time (ortho-para conversion was relatively slow) and the hyperpolarization was produced continuously, the following procedure was convenient for determining A^{HP} and A^{TH}. After adding parahydrogen, shaking and insertion the sample tube into the NMR magnet, the initial polarization was read in a single scan experiment. The measurement moment was set to be the time 0 point. By doing this we minimized effects coming from the sudden insertion of the sample to the high magnetic field, since we effectively destroy the polarization by the applied RF pulse. During the following 15 s, the reaction was let to produce a new portion of hyperpolarized alkenes in the high magnetic field that was detected by the next single-scan acquisition. Integration of signals showing the hyperpolarization in the second acquisition were used as A^{HP} values. The reaction was monitored by NMR till the moment when no hyperpolarization effects for both HCAT-alkyne-H_2 and the product alkene were observed. At this moment, we assume that para-H_2 was completely converted into normal H_2. Depending on the substrate, reaching this moment took 80 to 360 s as counted from the detection of the first spectrum. Knowing the time last from the shake till the detection of the purely thermal signals (Δ, s) and the amplitude of the thermal signals ($A^{\Sigma TH}$), it was possible to estimate the initial thermal signal accumulation rate $r_A = A^{\Sigma TH}/\Delta$. Taking into account that 15 s is the reaction time to produce the hyperpolarized alkene with amplitude A^{HP}, it was possible to estimate the amplitude of the thermally polarized signals $A^{TH} = r_A \times 15$ s. Since the reaction was not very fast, the linear approximation should give the reasonable estimates, which was confirmed in experiments with normal H_2. As the 15 s reaction time was not optimized for each alkene, this procedure provides the lower limit of enhancement factors. In principle, optimization of the time frame could allow repeated scan accumulation of the hyperpolarized signals. A very precise determination and optimization of reaction conditions are out of the scope of this initial study. Nevertheless, the currently provided enhancement factors characterize the potential of HCAT: at least two orders of magnitude signal enhancements are observable.

Table S1. Estimated enhancements of alkene signals in metal-free hydrogenations of alkynes **1-5**.

Alkene[a]	Signal position (δ),[b] ppm	Enhancement, natural HCAT	Enhancement, ^{15}N-HCAT
**P1**	5.52	160	100
**P2**	5.61	70	80
**P3**	5.81	210	110
**P4**	5.34	50	50
**P5**	6.44	no effect	no effect

[a]For alkene structures see Scheme S3, top. [b]The signals correspond to hydrogens marked with "*" in Scheme 3.

3 Characterization of intermediates using PHIP

3.1 ^{1}H, ^{11}B and ^{15}N NMR spectra of reaction intermediates

3.1.1 Hydrogenation of 1

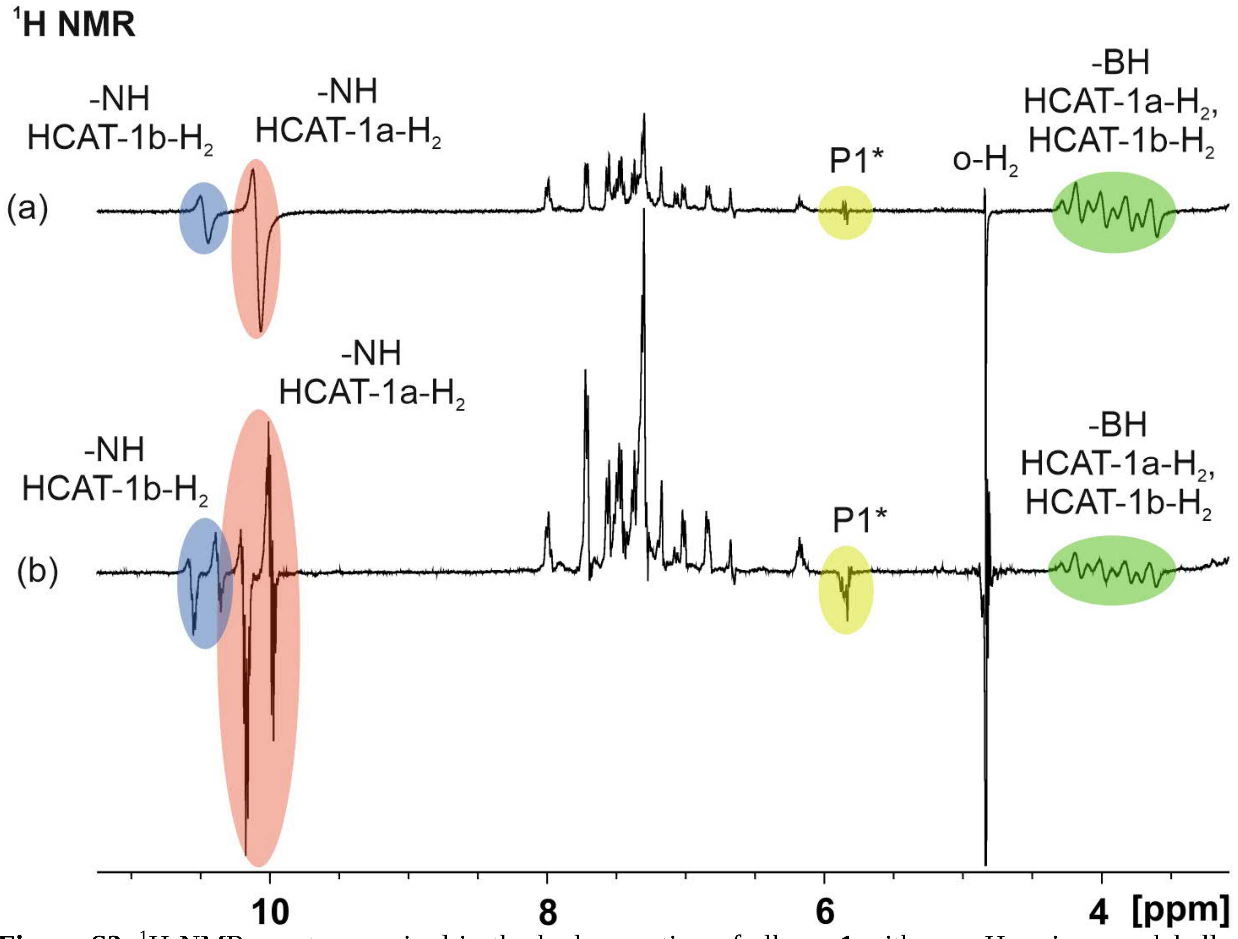

Figure S3. ^{1}H NMR spectra acquired in the hydrogenation of alkyne **1** with para-H_2 using non-labelled HCAT (a) and ^{15}N-labelled HCAT (b) catalysts. Both spectra were recorded in the very beginning of reaction immediately after addition of para-H_2 at room temperature. Hyperpolarization effects are visible mostly for HCAT-alkyne-H_2 intermediates, -NH/-BH group ^{1}H NMR signals and ortho-H_2. The alkene product **P1** is only slightly hyperpolarized due to short time passed after the reaction beginning. The position of the hyperpolarized ^{1}H nuclei in the alkenes is shown with "*" in Scheme S3. Overall, positions of signals of interest are highlighted using transparent ovals.

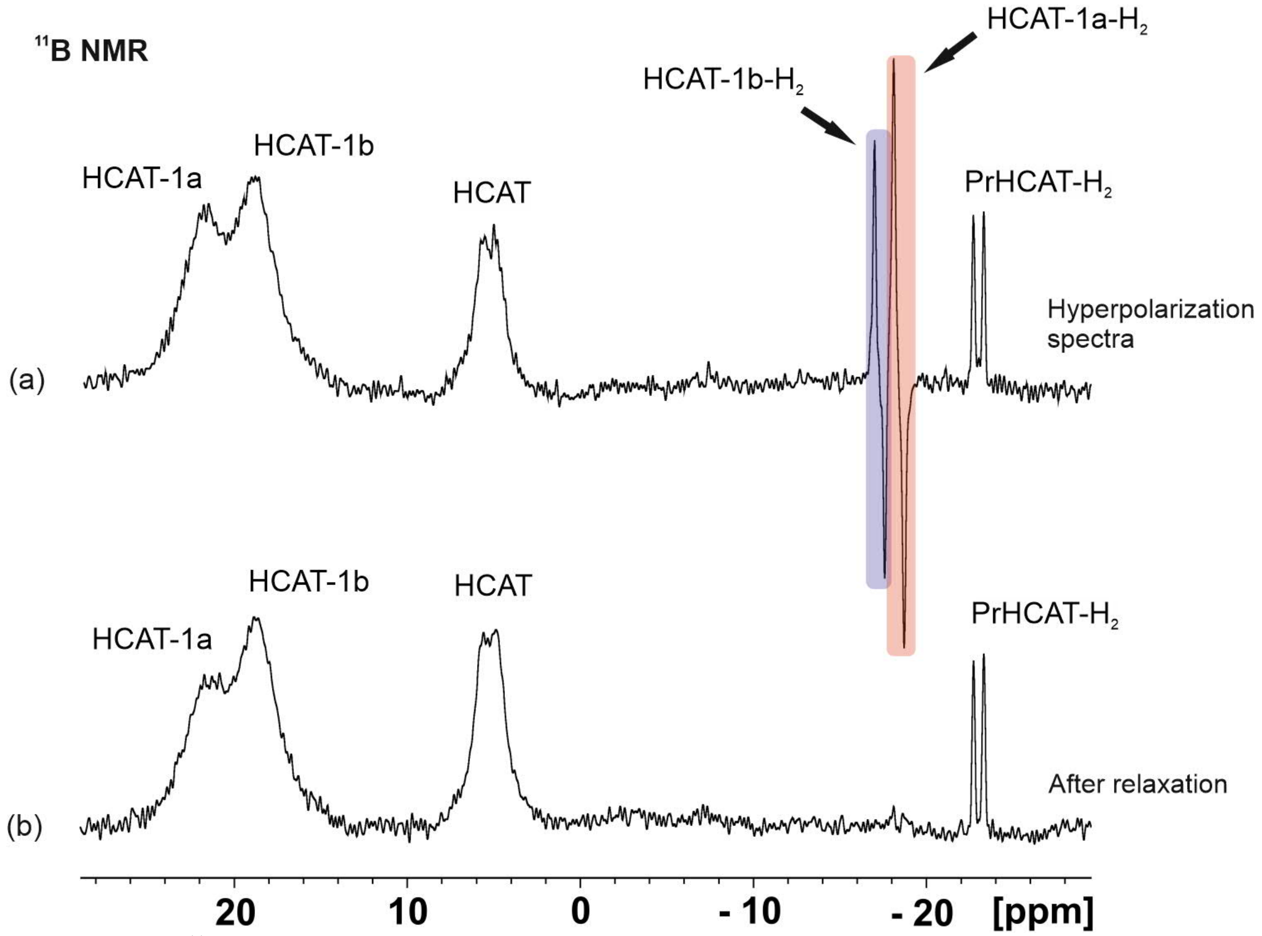

Figure S4. (a) ^{11}B NMR spectra acquired after introducing a fresh portion of para-H_2 into the mixture of HCAT catalyzed hydrogenation reaction of **1** (a) and *ca.* 100 s seconds later after conversion of para-H_2 and return to thermal equilibrium (b). Antiphase signals of HCAT-alkyne-H_2 intermediates are highlighted using transparent rectangles. The spectra reveal the presence of small amounts of PrHCAT-H_2 left after HCAT synthesis (Scheme 1). The spectra were recorded using 64 scan accumulations.

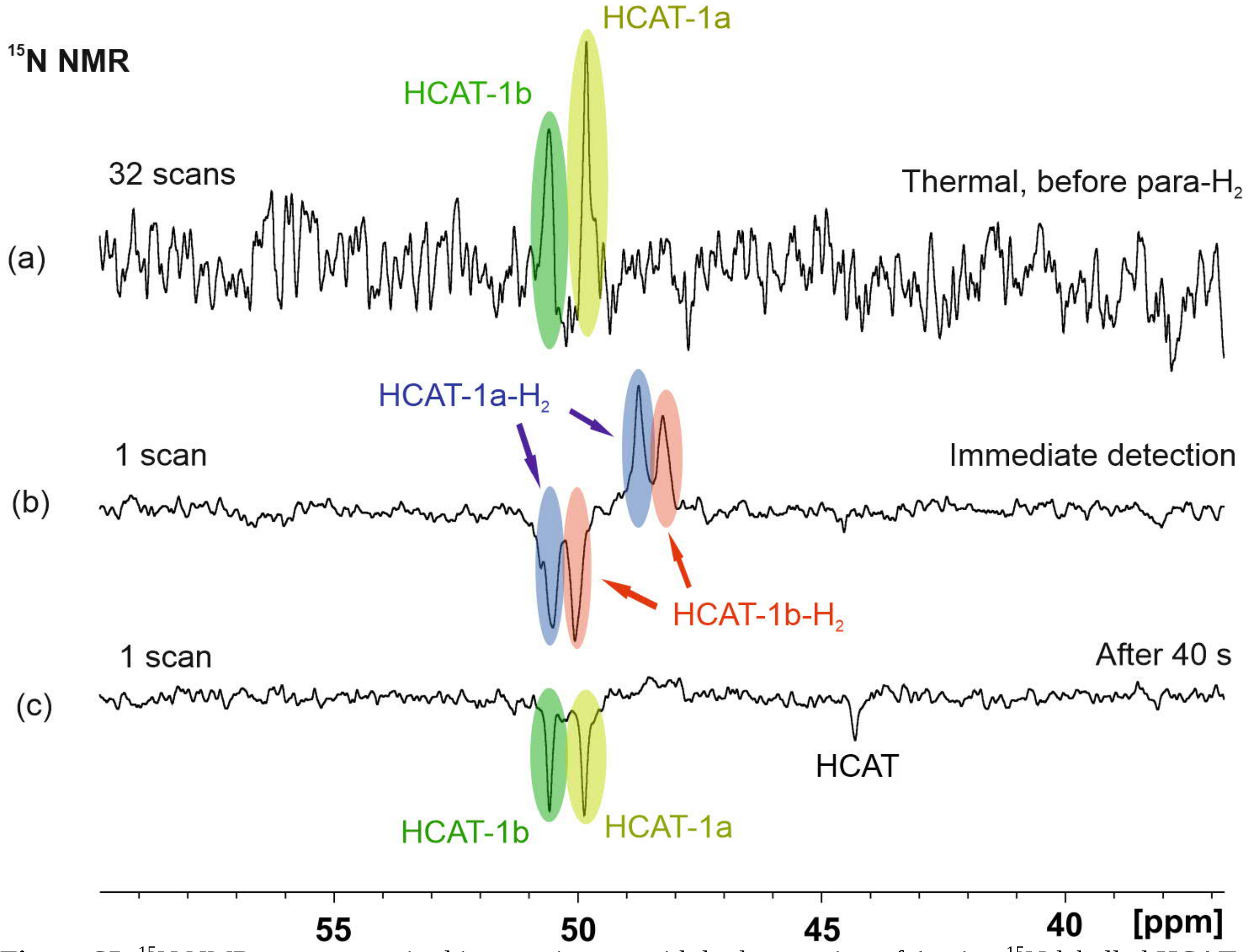

Figure S5. ^{15}N NMR spectra acquired in experiments with hydrogenation of **1** using ^{15}N-labelled HCAT. (a) A spectrum measured using 32 scan accumulations after preparation of HCAT-alkyne mixture for hydrogenation experiments but before actual addition of para-H_2. Spectra (b) and (c) are measured in 1 scan immediately after addition of para-H_2 and 40 s later, respectively. The signals corresponding to different compounds are highlighted using colored transparent ovals for a better representation. It is visible that the distribution of spontaneously hyperpolarized products is changing with time in ^{15}N NMR, making it possible to detect important HCAT-alkyne-H_2 intermediates, HCAT-alkyne adducts and HCAT itself.

3.1.2 Hydrogenation of 2

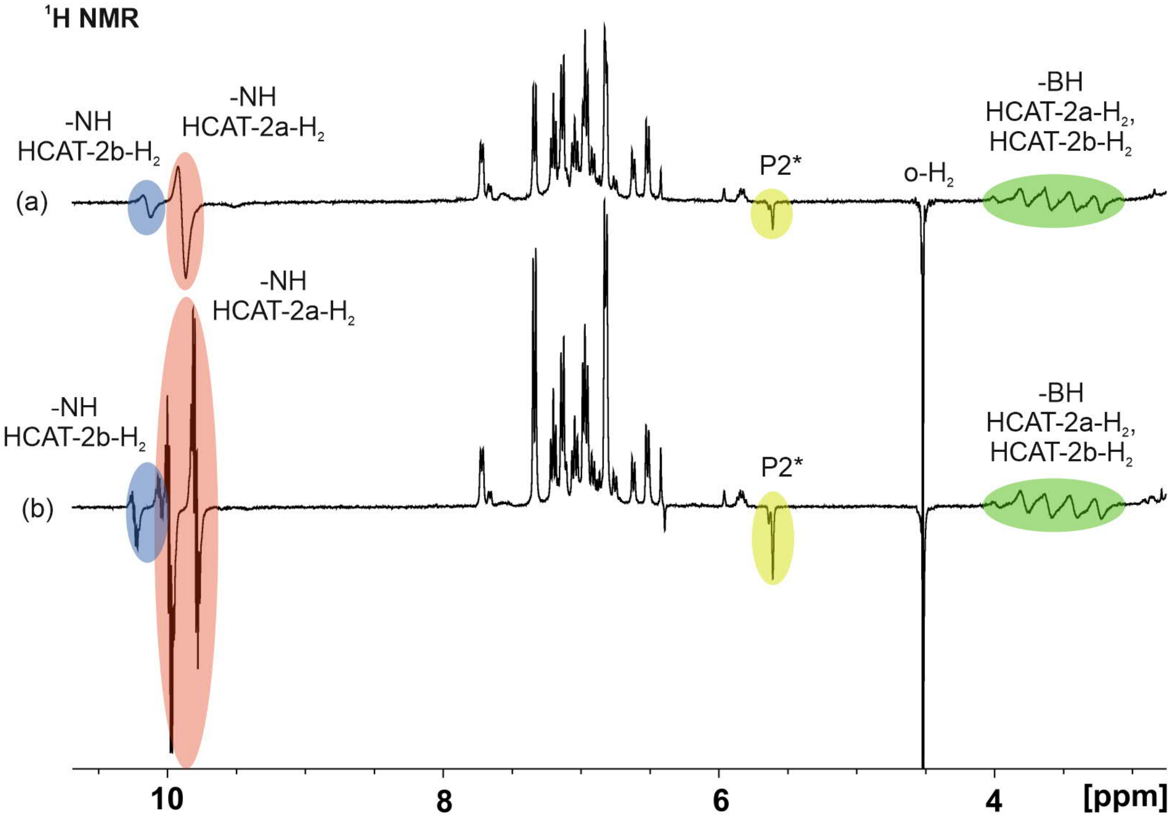

Figure S6. ^{1}H NMR spectra acquired in the hydrogenation of alkyne **2** with para-H_2 using non-labelled HCAT (a) and ^{15}N-labelled HCAT (b) catalysts. Both spectra were recorded after addition of para-H_2 at room temperature. Hyperpolarization effects are visible for HCAT-alkyne-H_2 intermediates, -NH/-BH group ^{1}H NMR signals, ortho-H_2, and the alkene product **P2**. The position of the hyperpolarized ^{1}H nuclei in the alkenes is shown with “*” in Scheme S3. Overall, positions of signals of interest are highlighted using transparent ovals.

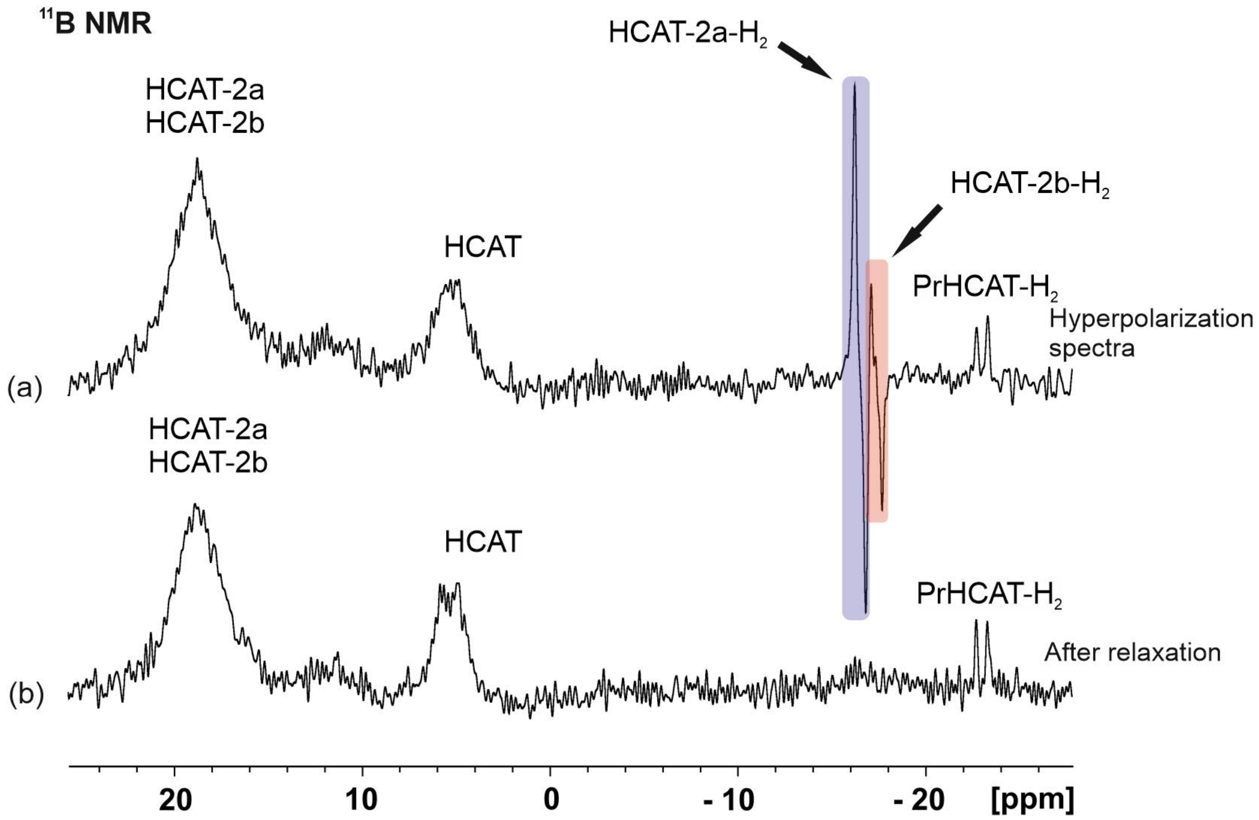

Figure S7. (a) ^{11}B NMR spectra acquired after introducing a fresh portion of para-H_2 into the mixture of HCAT catalyzed hydrogenation reaction of **2** (a) and *ca.* 90 s seconds later after conversion of para-H_2 and return to thermal equilibrium (b). Antiphase signals of HCAT-alkyne-H_2 intermediates are highlighted using transparent rectangles. The spectra reveal the presence of small amounts of PrHCAT-H_2 left after HCAT synthesis (Scheme 1). The spectra were recorded using 16 scan accumulations.

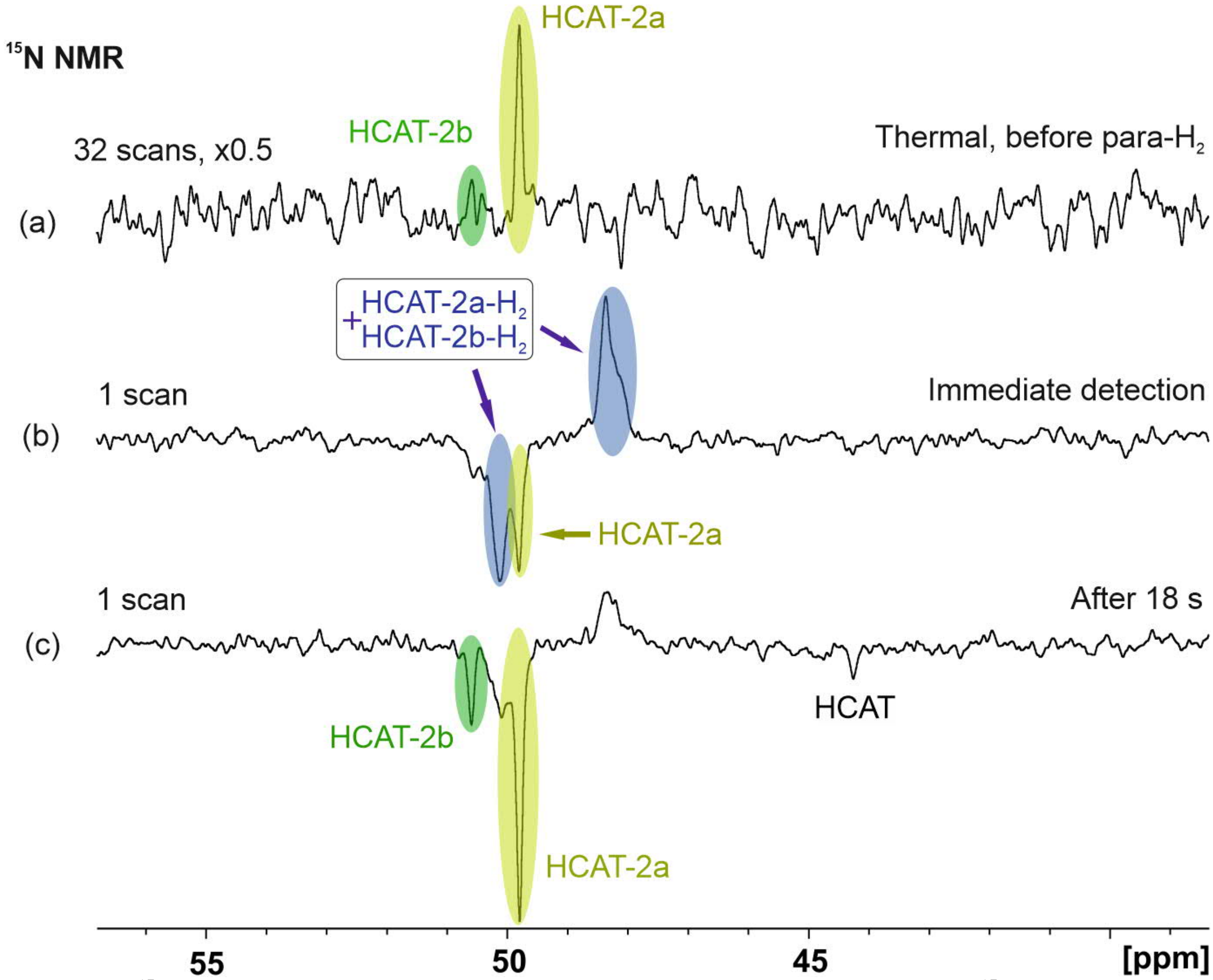

Figure S8. ^{15}N NMR spectra acquired in experiments with hydrogenation of **1** using ^{15}N-labelled HCAT. (a) A spectrum measured using 32 scan accumulations after preparation of HCAT-alkyne mixture for hydrogenation experiments but before actual addition of para-H_2. Spectra (b) and (c) are measured in 1 scan immediately after addition of para-H_2 and 18 s later, respectively. The signals corresponding to different compounds are highlighted using colored transparent ovals for a better representation. It is visible that the distribution of spontaneously hyperpolarized products is changing with time in ^{15}N NMR, making is possible to detect important HCAT-alkyne-H_2 intermediates, HCAT-alkyne adducts and HCAT itself.

3.1.3 Hydrogenation of 3

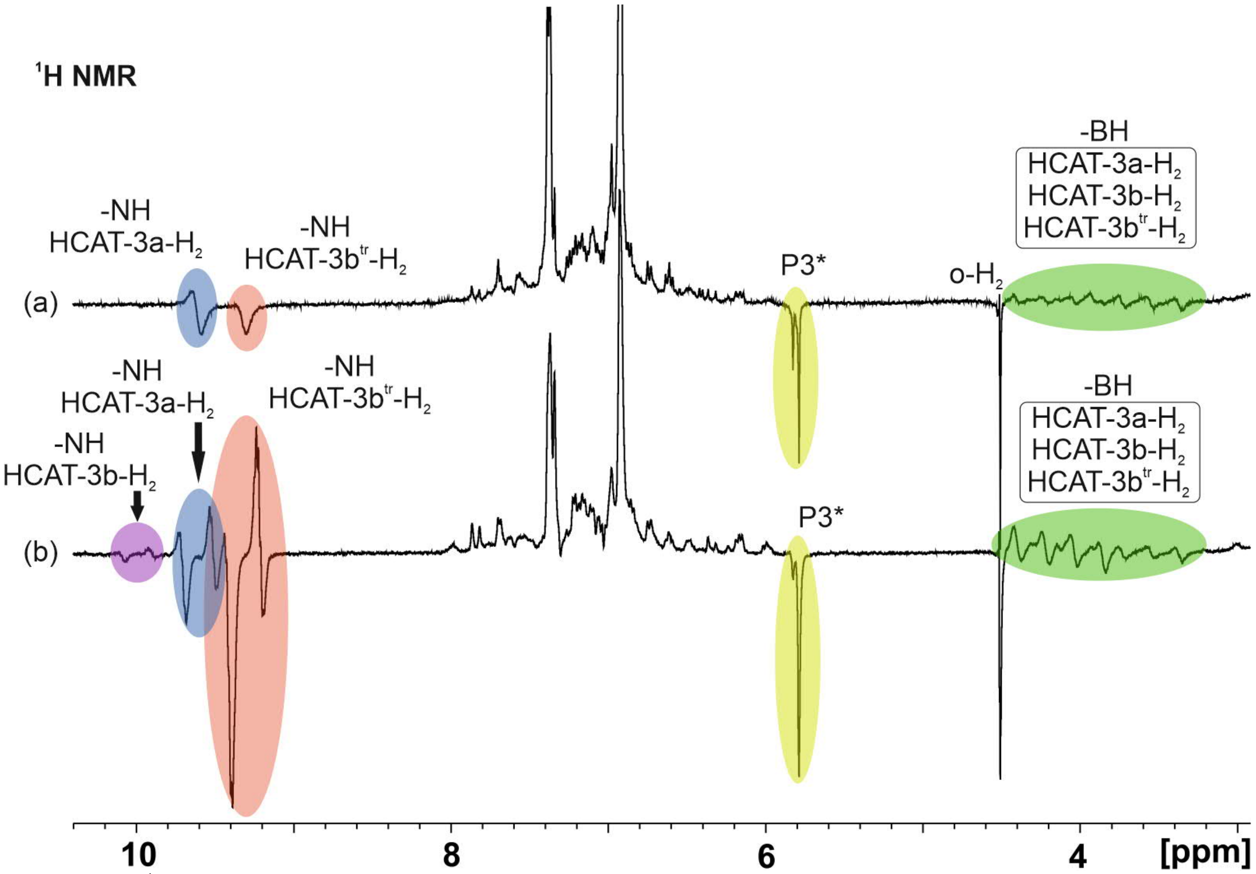

Figure S9. ^{1}H NMR spectra acquired in the hydrogenation of alkyne **3** with para-H_2 using non-labelled HCAT (a) and ^{15}N-labelled HCAT (b) catalysts. Both spectra were recorded after addition of para-H_2 at room temperature. Hyperpolarization effects are visible for HCAT-alkyne-H_2 intermediates, -NH/-BH group ^{1}H NMR signals, ortho-H_2, and the alkene product **P3**. The position of the hyperpolarized ^{1}H nuclei in the alkenes is shown with "*" in Scheme S3. Overall, positions of signals of interest are highlighted using transparent ovals. For the mechanism of formation of HCAT-3b^{tr}-H_2 and other underlying processes in this reaction see Scheme S4.

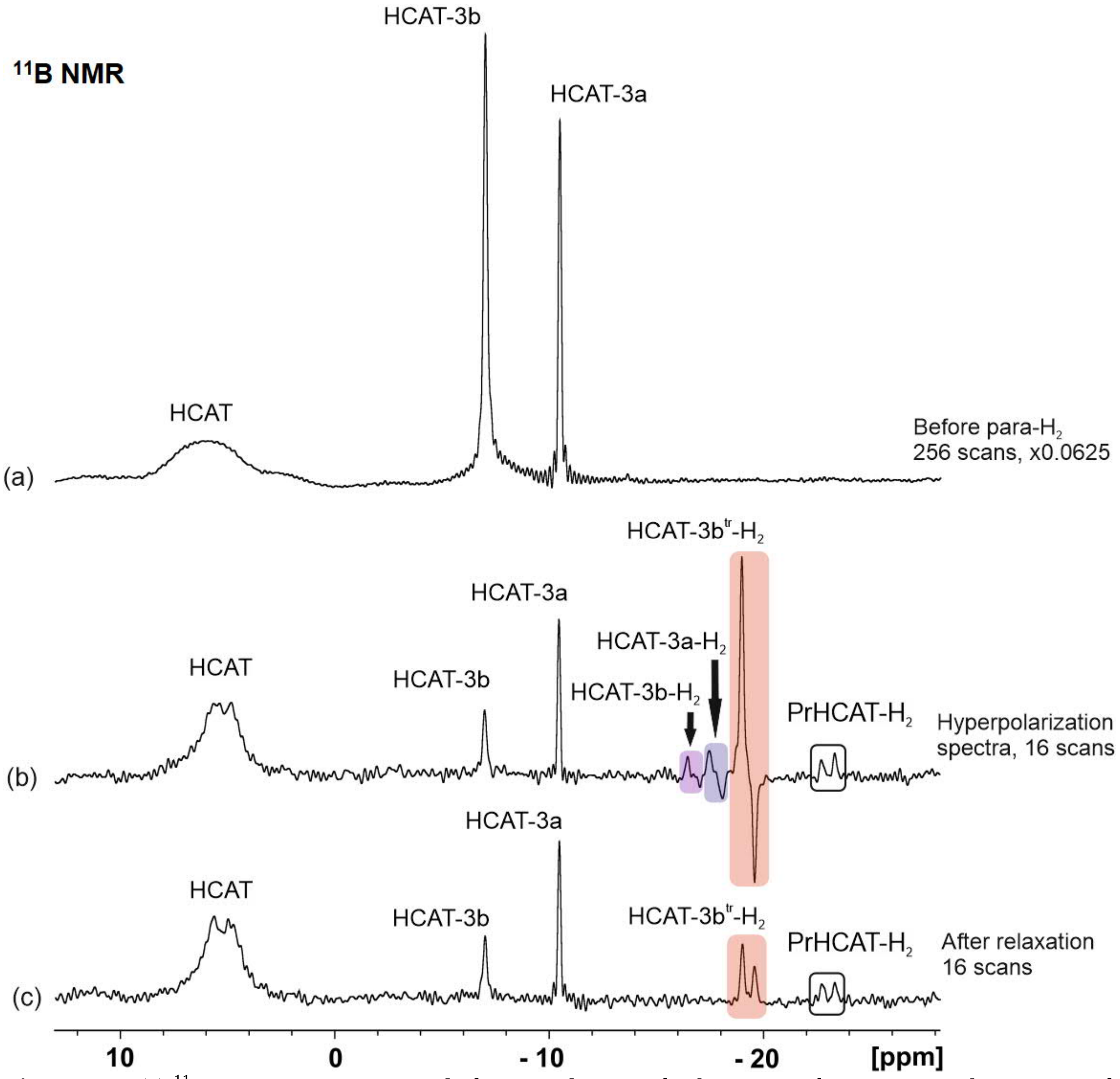

Figure S10. (a) ^{11}B NMR spectra acquired after introducing a fresh portion of para-H_2 into the mixture of HCAT catalyzed hydrogenation reaction of **3** (a) and *ca.* 140 s seconds later after conversion of para-H_2 and return to thermal equilibrium (b). Antiphase signals of HCAT-alkyne-H_2 intermediates are highlighted using transparent rectangles. The spectra reveal the presence of small amounts of PrHCAT-H_2 left after HCAT synthesis (Scheme 1). The spectra were recorded using 16 scan accumulations. For the mechanism of formation of HCAT-3b^{tr}-H_2 and other underlying processes in this reaction see Scheme S4.

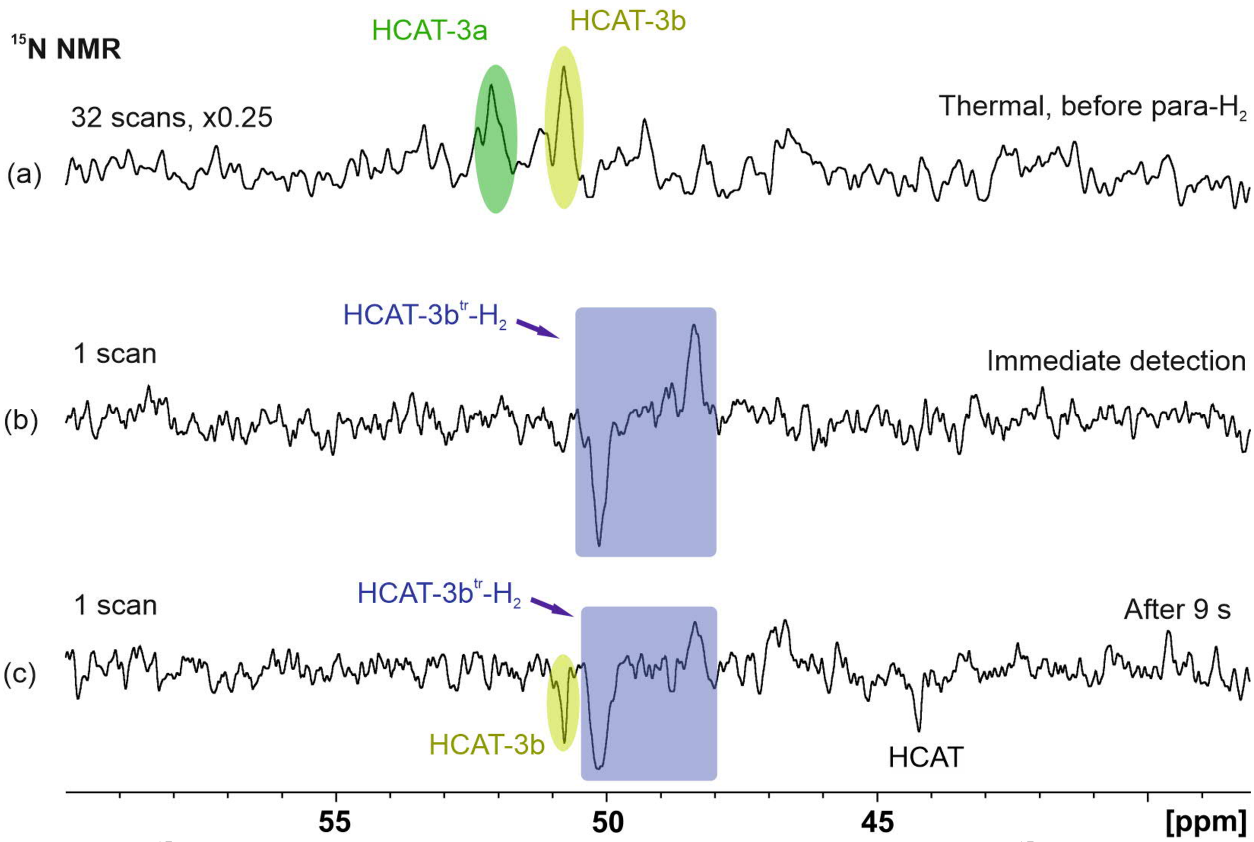

Figure S11. ^{15}N NMR spectra acquired in experiments with hydrogenation of **3** using ^{15}N-labelled HCAT. (a) A spectrum measured using 32 scan accumulations after preparation of HCAT-alkyne mixture for hydrogenation experiments but before actual addition of para-H_2. Spectra (b) and (c) are measured in 1 scan immediately after addition of para-H_2 and 9 s later, respectively. The signals corresponding to different compounds are highlighted using colored transparent ovals and rectangles for a better representation. It is visible that the distribution of spontaneously hyperpolarized products is changing with time in ^{15}N NMR, making is possible to detect important HCAT-alkyne-H_2 intermediates, HCAT-alkyne adducts and HCAT itself. For the mechanism of formation of HCAT-3b^{tr}-H_2 and other underlying processes in this reaction see Scheme S4.

3.1.4 Hydrogenation of 4

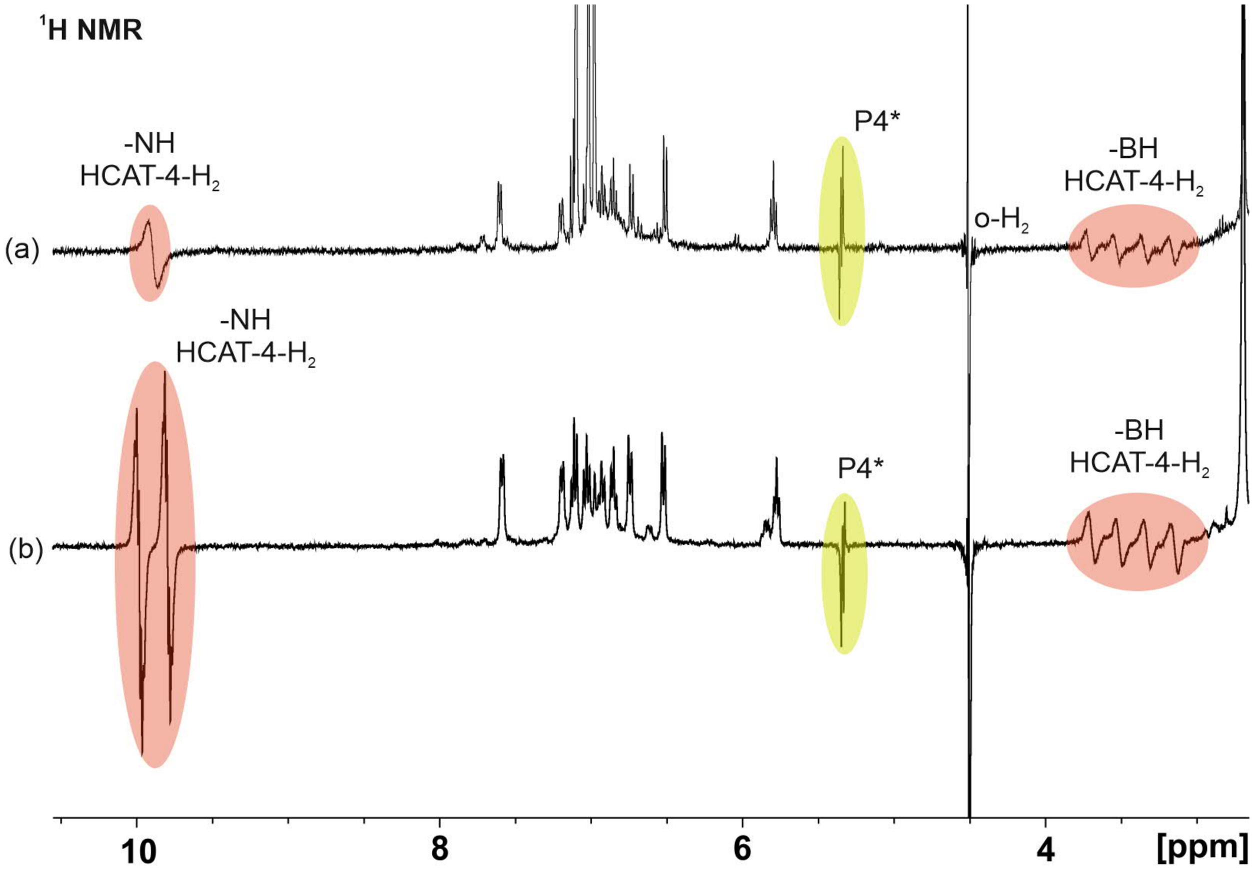

Figure S12. ^{1}H NMR spectra acquired in the hydrogenation of alkyne **4** with para-H_2 using non-labelled HCAT (a) and ^{15}N-labelled HCAT (b) catalysts. Both spectra were recorded after addition of para-H_2 at room temperature. Hyperpolarization effects are visible for HCAT-alkyne-H_2 intermediates, -NH/-BH group ^{1}H NMR signals, ortho-H_2, and the alkene product **P4**. The position of the hyperpolarized ^{1}H nuclei in the alkenes is shown with "*" in Scheme S3. Overall, positions of signals of interest are highlighted using transparent ovals.

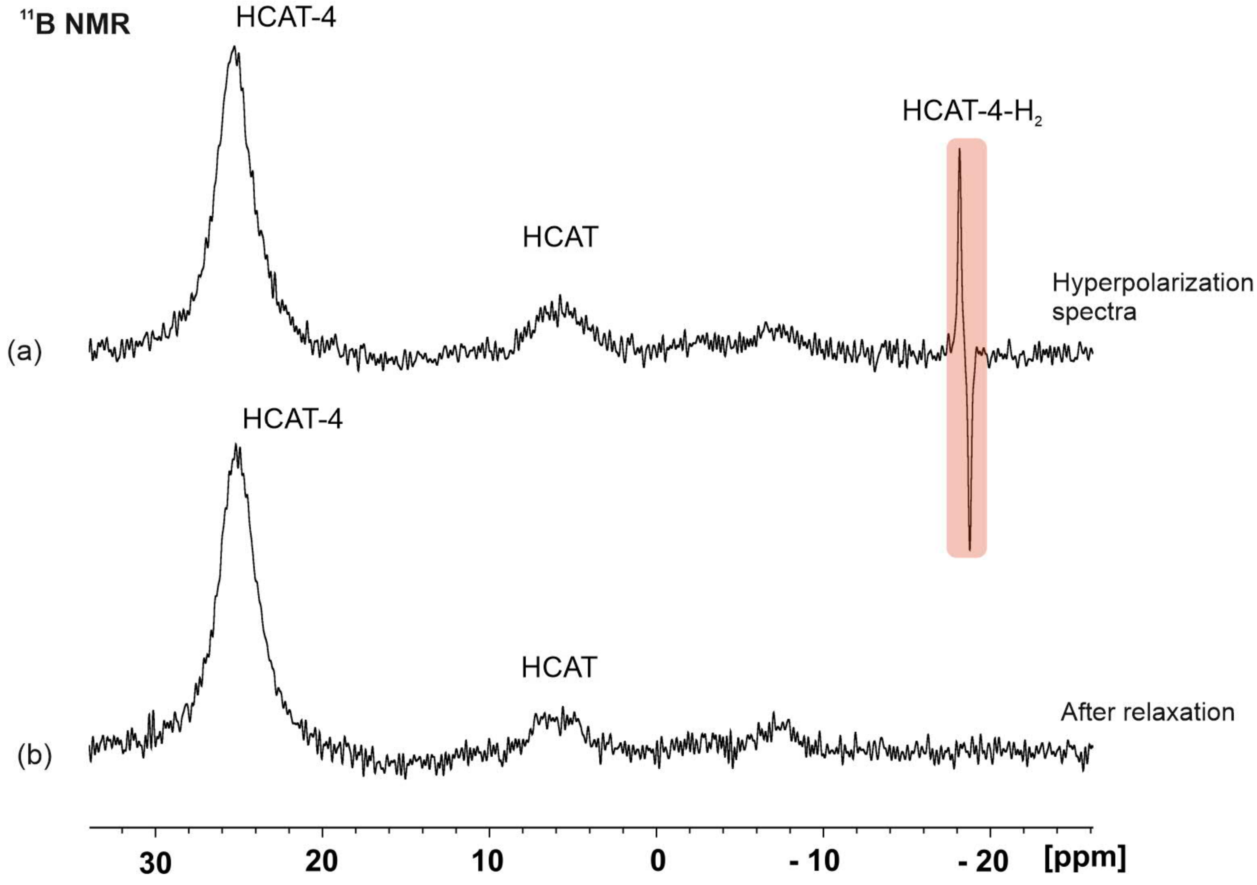

Figure S13. (a) ^{11}B NMR spectra acquired after introducing a fresh portion of para-H_2 into the mixture of HCAT catalyzed hydrogenation reaction of **4** (a) and *ca.* 90 s seconds later after conversion of para-H_2 and return to thermal equilibrium (b). Antiphase signal of HCAT-4-H_2 intermediate is highlighted using transparent rectangle. The spectra were recorded using 16 scan accumulations.

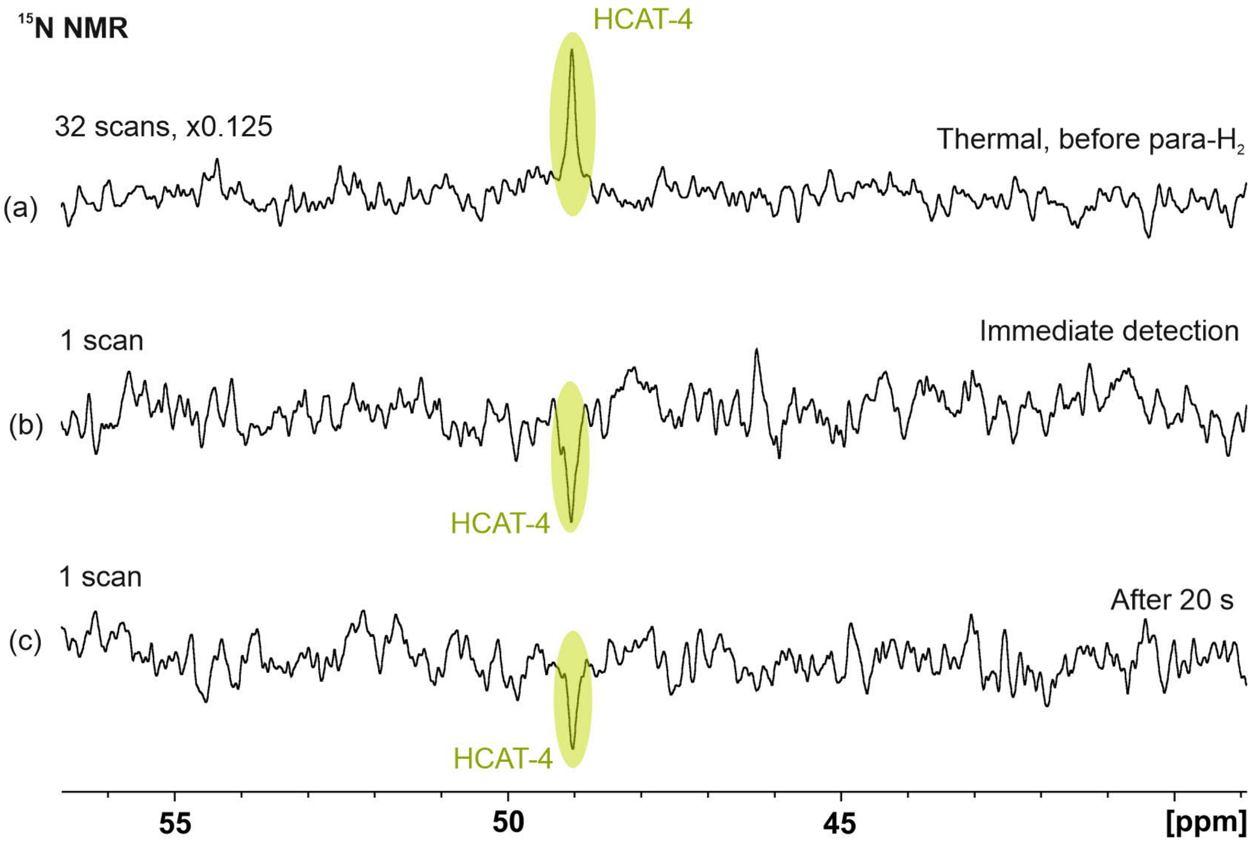

Figure S14. ^{15}N NMR spectra acquired in experiments with hydrogenation of **4** using ^{15}N-labelled HCAT. (a) A spectrum measured using 32 scan accumulations after preparation of HCAT-alkyne mixture for hydrogenation experiments but before actual addition of para-H_2. Spectra (b) and (c) are measured in 1 scan immediately after addition of para-H_2 and 20 s later, respectively. The signals corresponding to different compounds are highlighted using colored transparent ovals for a better representation. It is visible that the distribution of spontaneously hyperpolarized products is changing with time in ^{15}N NMR, making is possible to detect HCAT-4 adduct with one scan. HCAT-4-H_2 was not detected likely due to its low concentration.

3.1.5 Hydrogenation of 5

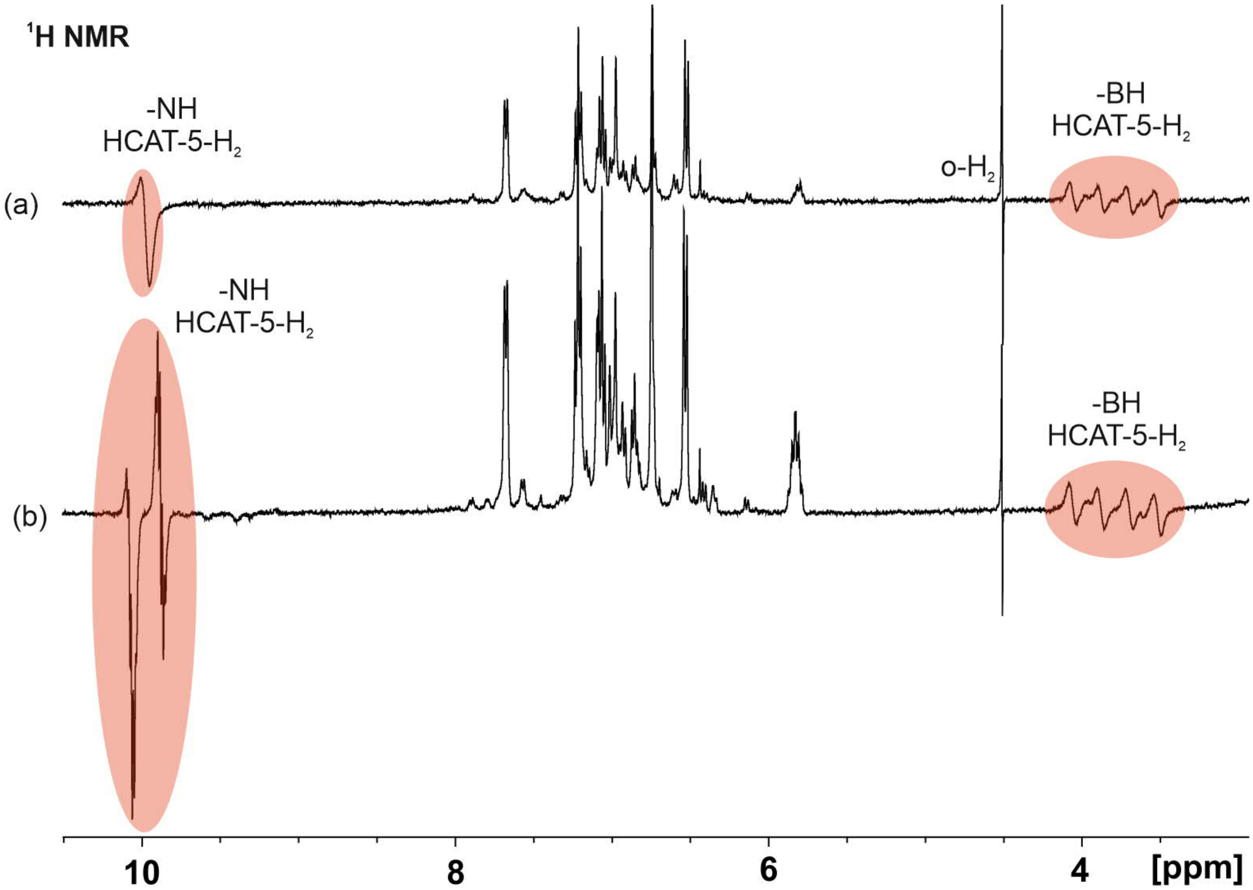

Figure S15. ^{1}H NMR spectra acquired in the hydrogenation of alkyne **5** with para-H_2 using non-labelled HCAT (a) and ^{15}N-labelled HCAT (b) catalysts. Both spectra were recorded after addition of para-H_2 at room temperature. Hyperpolarization effects are visible for HCAT-alkyne-H_2 intermediates, -NH/-BH group ^{1}H NMR signals, and ortho-H_2. Alkene product **P5** did not reveal any hyperpolarization effects. The position of the hyperpolarized ^{1}H nuclei in the alkenes is shown with “*” in Scheme S3. Overall, positions of signals of interest are highlighted using transparent ovals.

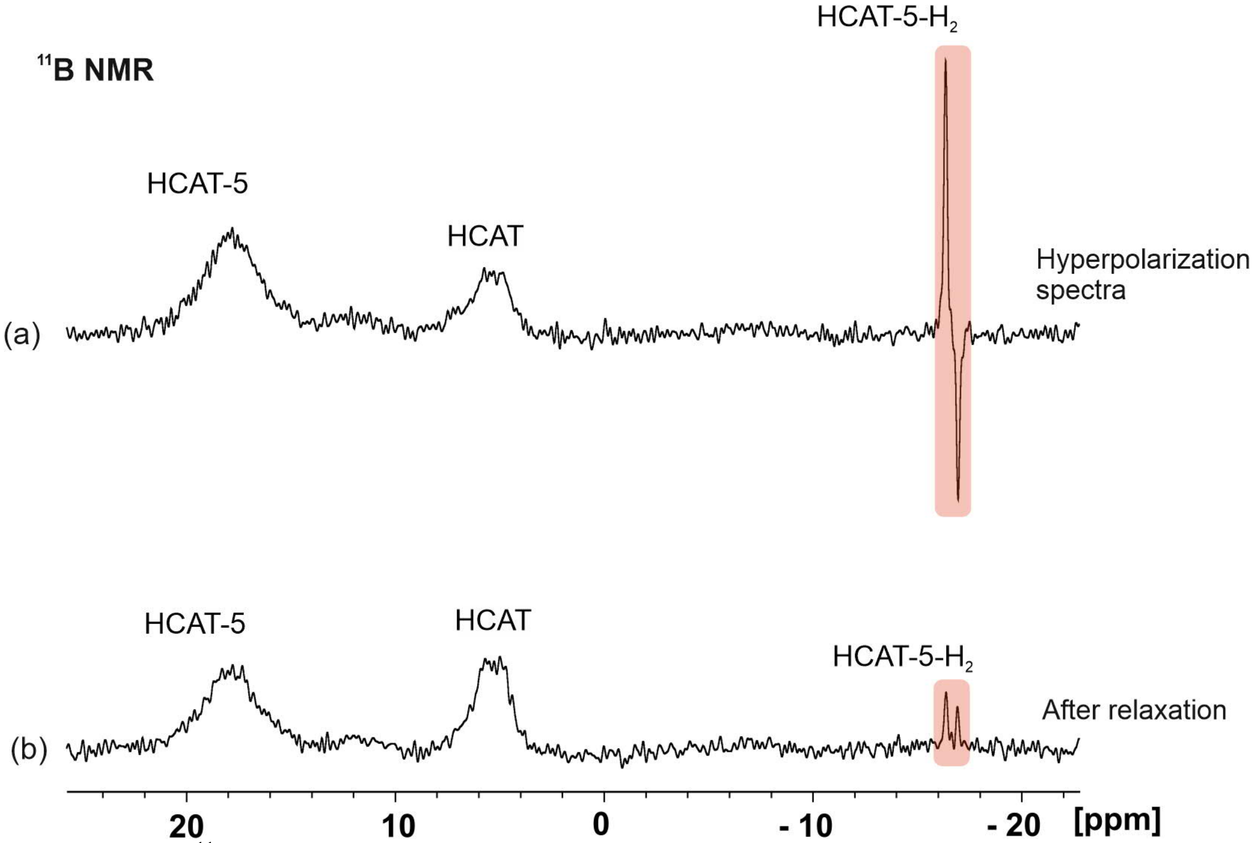

Figure S16. (a) ^{11}B NMR spectra acquired after introducing a fresh portion of para-H_2 into the mixture of HCAT catalyzed hydrogenation reaction of **5** (a) and *ca*. 90 s seconds later after conversion of para-H_2 and return to thermal equilibrium (b). Antiphase signal of HCAT-5-H_2 intermediate is highlighted using transparent rectangle. The spectra were recorded using 16 scan accumulations.

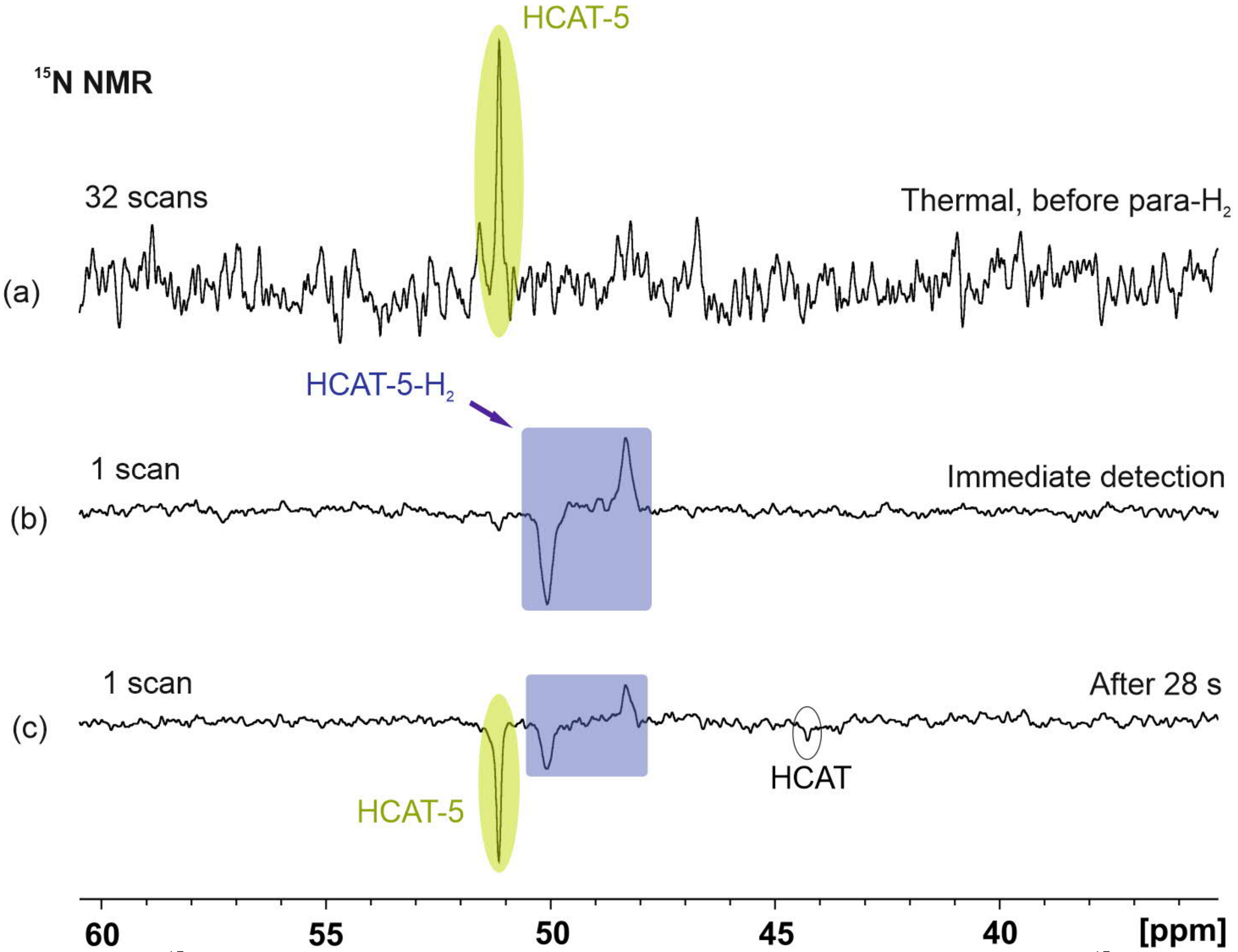

Figure S17. ^{15}N NMR spectra acquired in experiments with hydrogenation of **5** using ^{15}N-labelled HCAT. (a) A spectrum measured using 32 scan accumulations after preparation of HCAT-alkyne mixture for hydrogenation experiments but before actual addition of para-H_2. Spectra (b) and (c) are measured in 1 scan immediately after addition of para-H_2 and 28 s later, respectively. Signals corresponding to different compounds are highlighted using colored transparent ovals and rectangles for a better representation. It is visible that the distribution of spontaneously hyperpolarized products is changing with time in ^{15}N NMR, making is possible to detect important HCAT-5-H_2 intermediate, HCAT-5 adduct and HCAT in one scan.

3.2 Signal enhancements for HCAT-alkyne-H_2 intermediates and HCAT-alkyne adducts

Since in most of the cases it was impossible to observe thermal signals of HCAT-alkyne-H_2 and HCAT-alkyne species due to low concentrations, their signal enhancements were estimated by comparison of the hyperpolarized signal intensities to the noise level intensity in the corresponding ^{1}H, ^{15}N and ^{11}B NMR spectra, assuming that the thermal signals cannot be stronger than the noise level because they were not observable. This procedure gives only a rough idea about the actual signal enhancement, as it provides only the lower limit of what can be observed in the experiments with hyperpolarization. The following tables show these numbers for the hydrogenations with ^{15}N-labelled (Table S2) and non-labelled (Table S3) HCAT.

Table S2. Estimated signal enhancements of ^{15}N-labelled intermediates in HCAT catalyzed metal-free hydrogenations of alkynes **1-5**.

Compound[a]	Enhancement ^{1}H	Enhancement ^{15}N	Enhancement ^{11}B[b,c]
HCAT-1a-H_2	>270	>10	>40[b]
HCAT-1b-H_2	>70	>10	>30[b]
HCAT-2a-H_2	>250	>10	>20[c]
HCAT-2b-H_2	>50	>10	>10[c]
HCAT-3a-H_2	>60	-	>5[c]
HCAT-3b-H_2	>10	-	>5[c]
HCAT-3b^{tr}-H_2	50	>15	5[c]
HCAT-4-H_2	>100	-	>15[c]
HCAT-5-H_2	200	>10	-[d]
HCAT-1a	-	>40	-
HCAT-1b	-	>40	-
HCAT-2a	-	>40	-
HCAT-2b	-	>40	-
HCAT-3b	-	>10	-
HCAT-4	-	>10	-
HCAT-5	-	>10	-

[a]For structures of intermediates see Scheme 3; [b]64 scan ^{11}B NMR spectra were analyzed.; [c]16 scan ^{11}B NMR spectra were analyzed; [d]was not measured.

Table S3. Estimated signal enhancements of non-labelled intermediates in HCAT catalyzed metal-free hydrogenations of alkynes **1-5**.

Compound[a]	Enhancement ^{1}H	Enhancement ^{11}B[b,c]
HCAT-1a-H_2	>140	>10[b]
HCAT-1b-H_2	>40	>10[b]
HCAT-2a-H_2	>100	>15[b]
HCAT-2b-H_2	>25	>5[b]
HCAT-3a-H_2	>30	>5[b]
HCAT-3b-H_2	-	>5[b]
HCAT-3b^{tr}-H_2	>40	5[b]
HCAT-4-H_2	>30	>5[b]
HCAT-5-H_2	>70	5[c]

[a]For structures of intermediates see Scheme S3; [b]64 scans ^{11}B NMR spectra were analyzed; [c]16 scans ^{11}B NMR spectra were analyzed.

4 Ruling out coherent mixing as a mechanism of one-hydrogen hyperpolarization in HCAT catalyzed hydrogenations

Herein, we discuss arguments against efficiency of the coherent mixing mechanism in our experiments. In principle, this mechanism can be also responsible for one-hydrogen hyperpolarization in PHIP. Originally formulated for explaining hyperpolarization of aldehydes in hydroformylation reactions by Permin and Eisenberg,[S9] it was used to describe similar effects, for instance, in water ligand exchange[S10] and for the so-called SWAMP[S11] (surface waters are magnetized by parahydrogen) effect.

The original one-hydrogen mechanism suggested by Permin and Eisenberg is based on the fact that in the case of formation a relatively strongly-coupled spin system out of the

parahydrogen originating proton pair, the parahydrogen singlet averaged over the formation time can lead to the single-spin polarizations of the parahydrogen originating protons in reaction intermediates. In the case of weakly-coupled systems this effect is not efficient. A good educational example is published, for instance in Figure 3c of Ref. S7. Bigger the ratio of chemical shift difference of the protons ($\Delta\delta$) to the J-coupling constant between them (J), lower the amplitude of the net single spin polarization revealed by $\pi/2$-pulse. In our case, that protons form weakly-coupled AX spin-system (typical chemical shift difference $\Delta\delta$ is 2500 Hz, and J-coupling constants J are about 14 Hz; $2*\Delta\delta/J = 356$, therefore $\Delta\delta >> J/2$), thus, generally, we can expect that the original mechanism by Permin and Eisenberg is not efficient. Specifically, this mechanism can be ruled out by noting that reaction averaging/state-projection in the coherent mixing must lead to the positive polarization of the NH group protons in HCAT-alkyne-H_2 intermediates as the J-coupling constant is positive based on the phase of the antiphase pattern. Figure S18 shows simulated PASADENA spectra for AX spin system with the chemical shift difference and the J-coupling constant typical for HCAT-alkyne-H_2 intermediates in our experiments (the ^{11}B-induced splitting is ignored). The phase of the antiphase signals in Figure S18a obtained by applying $\pi/4$-pulse correspond to the positive sign of the J-coupling constant and it correlates with the phase for the HCAT-alkyne-H_2 intermediates observed. At the same time, the sign of the net polarization corresponding to the downfield signal that models the NH proton in the intermediates should be positive with the positive J, as predicted in the simulation using a $\pi/2$-pulse rotation (Figure S18c). However, according to the catalytic cycle in Scheme 2, NH proton gains negative polarization and ends up in the alkene product after that. Our experiments clearly show the negative polarization of alkyne products. Therefore, this helps to rule out the coherent averaging mechanism quite strictly, since it should lead to the positive polarization according to the simulations. The simulation was performed in Matlab by numerical propagation of the initial parahydrogen density operator averaged over the reaction time. Similar results can be obtained by using, for instance, Eq. 26 from Ref. S7.

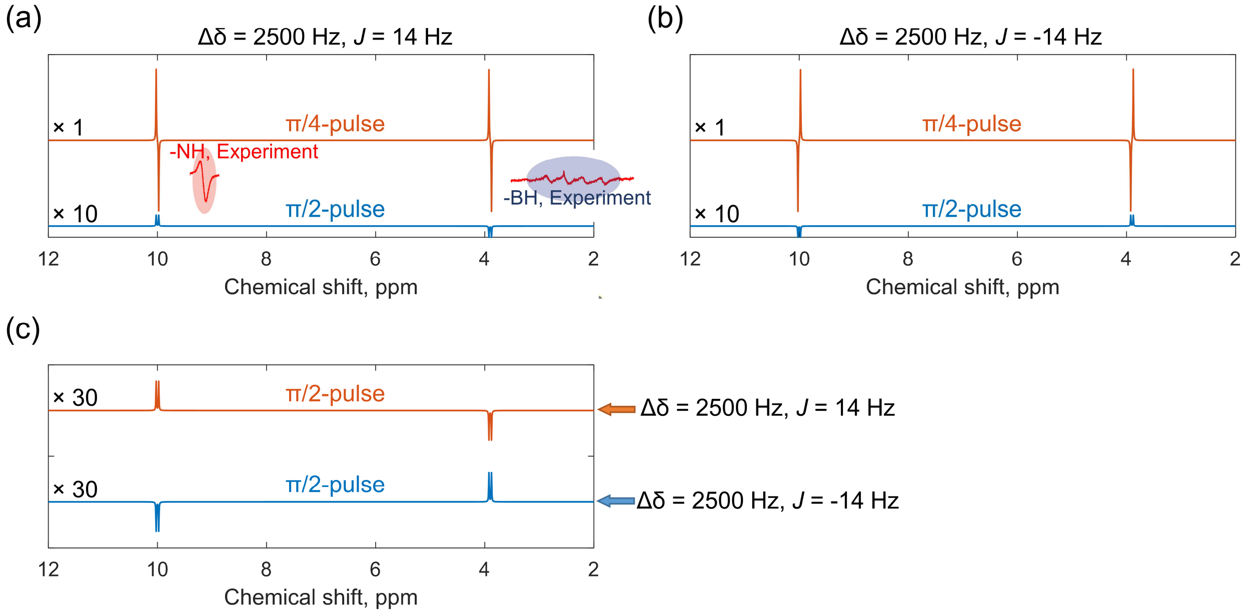

Figure S18. Numerical simulations of NMR signal patterns in PASADENA experiment for AX spin system. Typical parameters (Δδ, *J*) for HCAT-alkyne-H_2 intermediates were used. Traces (a) and (b) show the difference in phases of signals depending on the sign of the J-coupling constant *J*. The positive sign matches the experimental observations for NH and BH group protons. Traces (c) show comparison of signs of the net polarizations acquired by the individual protons as a function of *J* sign, indicating positive polarization for the NH group proton if the J-coupling constant is positive (upper trace).

5 Stereoisomerization in HCAT catalyzed hydrogenation of 3

In contrast to all other substrates, hydrogenation of **3** was accompanied by *cis*-*trans* isomerization leading to both *cis*- and *trans*- alkenes as products. The possibility of the both stereoisomers was reported for this substrate previously,[S1] while in this study using hyperpolarization effects we detected corresponding intermediates, which allowed proposing underlying mechanistic routes of this process (Scheme S4). Besides intermediates, only one, *gem*-TMS, vinylic hydrogen atom in the produced *cis*-alkene appeared to be hyperpolarized. Although the *trans*-product did not reveal any hyperpolarization effects, we assumed it was formed from HCAT-3b via the following plausible mechanism.

The observed stereoisomerization can be explained by the presence of TMS (trimethylsilane) substituent in the structure of **3**. It is known that silicon can stabilize positive charge at a carbon atom located in β position with respect to the silicon atom by the so-called beta-silicon effect or silicon hyperconjugation.[S12] Therefore, we can expect the formation of relatively stable carbocations with low barrier of ration that can serve as a driving force of the stereoisomerization. Route C in Scheme S4 shows elementary steps of this process. The hydroboration reaction leading to the formation of HCAT-alkyne adducts is highly *cis*-selective, which is justified by clear evidence described in many literature sources. Under the assumption that it is still *cis*-selective for the HCAT-alkyne adducts, without H_2 one should expect to detect

two *cis*-regioisomers after the interaction of HCAT and **3**, namely, HCAT-3a and HCAT-3b isomers. Indeed, this is what we do observed in the experiments. After addition of **3** to a solution of HCAT, ^{11}B NMR (Figure S10a) and ^{15}N NMR (Figure S11a) reveal a formation of the two HCAT-alkyne adducts. Importantly, a subsequent addition of para-H_2 leads to observation of three antiphase ^{11}B NMR doublets (Figure S10b) corresponding to three different HCAT-alkyne-H_2 intermediates. The use of ^{15}N-labelled HCAT also allowed observing three hyperpolarized -NH signals in ^{1}H NMR (Figure S9b) spectra. In the case of the non-labelled counterpart of HCAT, we saw only two intermediates since the amplitude of the third -NH signal was likely just too low to be visible (Figure S9a). These hyperpolarized intermediates can be assigned to HCAT-3a-H_2, HCAT-3b-H_2 and HCAT-3b^{tr}-H_2 intermediates (Scheme S4). ^{15}N NMR spectra measured immediately after the para-H_2 addition (Figure S11b) showed hyperpolarization of only HCAT-3b^{tr}-H_2, as it was in a higher concentration compared to other intermediates. The fact that addition of H_2 leads to the isomerized form of HCAT-3b-H_2 correlates with the formation of the TMS-stabilized beta-cation and rotation of the -CH_2TMS group in that carbocation (Route C). Looking at the structure of these species, rotation should be thermodynamically favorable, since bulky substituents, TMS and phenyl, will be further away from each other as a result of this process. According to our observations, HCAT-3b^{tr}-H_2 is also thermodynamically more sable as compared to HCAT-3b-H_2 as it is accumulated in quantities to be observable even without the strong hyperpolarization after relaxation to thermal equilibrium (see ^{11}B NMR, Figure S10c).

To sum up, Route A in Scheme S4 led to the formation of the hyperpolarized cis-alkene as a result of elimination from HCAT-3a-H_2. In ^{1}H NMR experiments, we observe hyperpolarization of the proton of this product located in geminal position with respect to TMS group. The hyperpolarization of the proton located in geminal position with respect to phenyl was not observed because of slow elimination of the product from HCAT-3a-H_2 (Route B) as compared to the lifetime of the hyperpolarization. In contrast, Route C led to *cis-trans* isomerization due to the stabilizing beta-effect of TMS. In principle, this route could lead to the formation of the hyperpolarized trans-alkene, but reaction rate was slow to let hyperpolarization survive before it is destroyed by the nuclear spin relaxation. The rate was much slower than in the case of Route A according to ^{1}H NMR.

Scheme S4. Mechanism of HCAT-catalyzed hydrogenation of **3** accompanied by *cis-trans* stereoisomerization

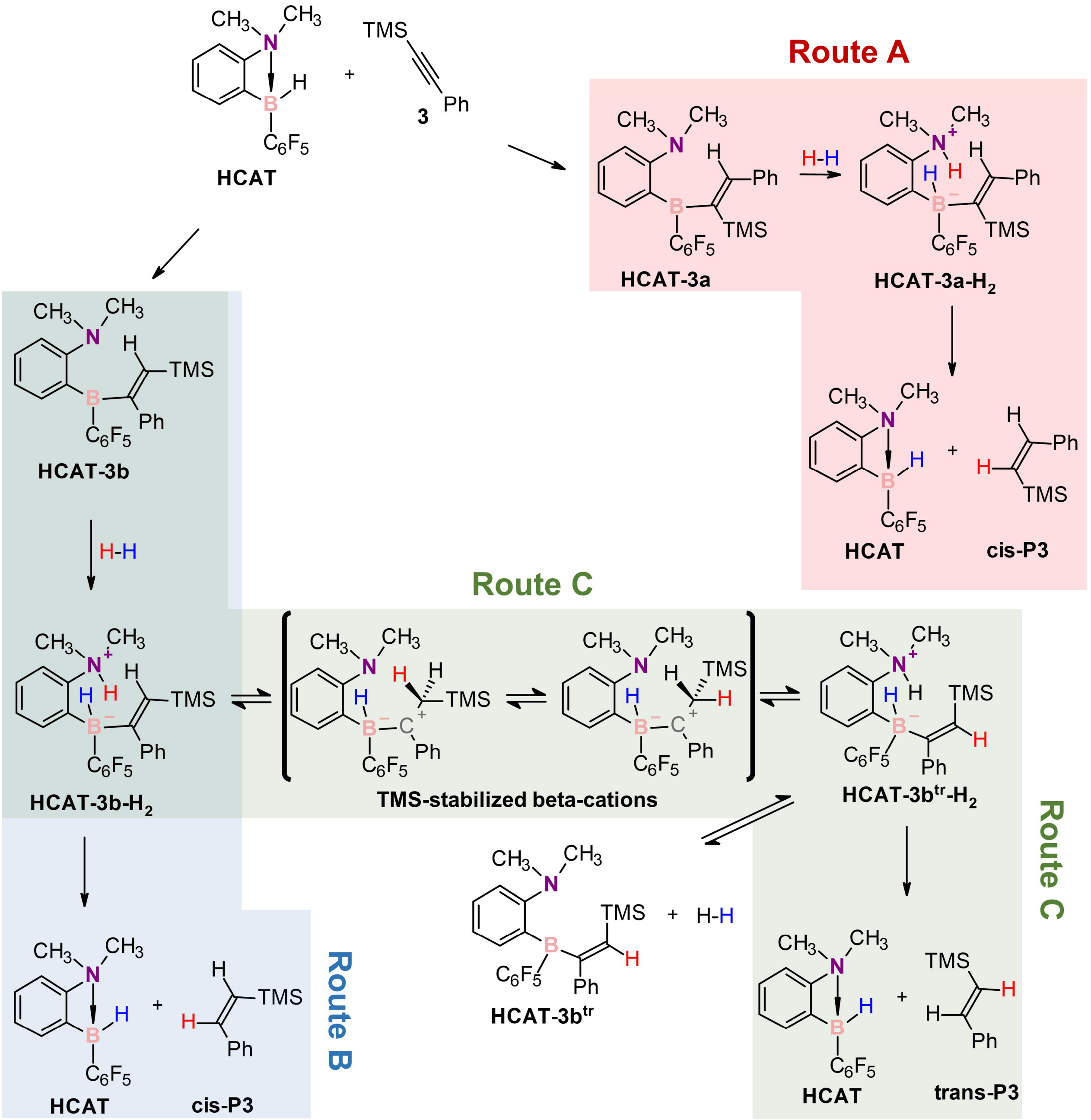

6 References

S1. K. Chernichenko, Á. Madarász, I. Pápai, M. Nieger, M. Leskelä and T. Repo, *Nat. Chem.*, 2013, **5**, 718-723.

S2. K. Chernichenko, M. Nieger, M. Leskela and T. Repo, *Dalton Trans.*, 2012, **41**, 9029-9032.

S3. B. A. Geller and L. S. Samosvat, *J. Gen. Chem. (USSR)*, 1960, **30**, 1594-1597.

S4. R. K. Kawade, D. B. Huple, R.-J. Lin and R.-S. Liu, *Chem. Commun.*, 2015, **51**, 6625-6628.

S5. K. Chernichenko, B. Kótai, M. Nieger, S. Heikkinen, I. Pápai and T. Repo, *Dalton Trans.*, 2017, **46**, 2263-2269.

S6. C. R. Bowers and D. P. Weitekamp, *J. Am. Chem. Soc.*, 1987, **109**, 5541-5542.

S7. C. R. Bowers, in *Encyclopedia of Nuclear Magnetic Resonance*, eds. D. M. Grant and R. K. Harris, Wiley, Chichester, 2002, vol. 9, ch. Chapter, pp. 750-769.

S8. M. G. Pravica and D. P. Weitekamp, *Chem. Phys. Lett.*, 1988, **145**, 255-258.

S9. A. B. Permin and R. Eisenberg, *J. Am. Chem. Soc.*, 2002, **124**, 12406-12407.

S10. M. Emondts, D. Schikowski, J. Klankermayer and P. P. M. Schleker, *ChemPhysChem*, 2018, **19**, 2614-2620.

S11. E. W. Zhao, R. Maligal-Ganesh, Y. Du, T. Y. Zhao, J. Collins, T. Ma, L. Zhou, T.-W. Goh, W. Huang and C. R. Bowers, *Chem*, 2018, **4**, 1387-1403.

S12. E. W. Colvin, in *Silicon in Organic Synthesis*, ed. E. W. Colvin, Butterworth-Heinemann, 1981, DOI: https://doi.org/10.1016/B978-0-408-10831-7.50009-3, pp. 15-20.
